# Causal effects of inflammatory protein biomarkers on inflammatory diseases

**DOI:** 10.1126/sciadv.abl4359

**Published:** 2021-12-08

**Authors:** Weronica E. Ek, Torgny Karlsson, Julia Höglund, Mathias Rask-Andersen, Åsa Johansson

**Affiliations:** Department of Immunology, Genetics and Pathology, Science for Life Laboratory, Uppsala University, Uppsala, Sweden.

## Abstract

Many circulating proteins are associated with the presence or severity of disease. However, whether these protein biomarkers are causal for disease development is usually unknown. We investigated the causal effect of 21 well-known or exploratory protein biomarkers of inflammation on 18 inflammatory diseases using two-sample Mendelian randomization. We identified six proteins to have causal effects on any of 11 inflammatory diseases (FDR < 0.05, corresponding to *P* < 1.4 × 10^–3^). IL-12B protects against psoriasis and psoriatic arthropathy, LAP-TGF-β-1 protects against osteoarthritis, TWEAK protects against asthma, VEGF-A protects against ulcerative colitis, and LT-α protects against both type 1 diabetes and rheumatoid arthritis. In contrast, IL-18R1 increases the risk of developing allergy, hay fever, and eczema. Most proteins showed protective effects against development of disease rather than increasing disease risk, which indicates that many disease-related biomarkers are expressed to protect from tissue damage. These proteins represent potential intervention points for disease prevention and treatment.

## Teaser

Many disease-associated protein biomarkers protect against disease development.

## INTRODUCTION

Inflammatory processes are associated with a large range of human diseases, including rheumatic diseases and allergies. Therefore, the role of inflammation in the development and progression of disease is of great scientific and public health interest. Protein biomarkers are measurable molecules that can have a prognostic value in patients, be used to diagnose disease, or indicate severity of disease. Today, a large number of measured plasma proteins have been identified as potential biomarkers for disease, which means that these proteins are differentially expressed (or only expressed) in patients with disease. In addition to being indicative of disease, these proteins may serve as potential drug targets or shed light into the pathogenesis of disease. However, the causal relation between a protein biomarker and its associated disease is commonly not known, in particular, the direction in which high/low levels of a protein could affect the risk of developing disease. Either the protein could be expressed in response to the disease, e.g., to protect from tissue damage, or the high expression of the protein could be the underlying factor that contributes to the development of disease.

Previous genome-wide association studies (GWAS) have identified a large number of genetic variants associated with the expression levels of protein biomarkers (*1*–*4*). These studies have opened up for the possibility to use genetic variants as instrumental variables (IVs) in Mendelian randomization (MR) analyses to unravel the direction of causality between disease and disease-related protein biomarkers (*3*, *5*, *6*). MR can be used to disentangle the effect that a protein exerts on the risk of developing disease from the reversed effect where disease development causes a change in protein abundance. MR can control both for reversed causation and for confounding by making use of variants that are associated with the exposure (i.e., amount of the circulating protein). As genetic variants are randomly assorted during the formation of gametes before conception, neither they can be affected by the outcome nor their effect on the outcome can be confounded by lifestyle or environmental factors in ethnically homogeneous and unrelated samples (*7*–*9*). It is, however, important to ensure that the variants are not horizontally pleiotropic. That is, they should not be associated with any confounding factor between the exposure and outcome and should only be associated with the outcome through the exposure (*10*).

The aim of this study was to identify inflammatory proteins that influence the risk of developing inflammatory diseases. To assess this, we undertook a two-sample MR approach to estimate the causal effect of 85 known, or suggested, protein biomarkers on 18 inflammatory diseases. For the two-sample design, we used two different population-based cohorts with no overlapping samples. In the first cohort, the Northern Sweden Population Health Study (NSPHS), we have already measured a large set of inflammatory protein biomarkers and performed high-coverage whole-genome sequencing (WGS) (*11*). As the second cohort, we used the UK Biobank (UKB), because it is the largest available cohort with genetic data and information on diagnoses for a large number of inflammatory diseases. We refrained from adopting publicly available GWAS summary statistics to avoid potential bias that may be introduced if covariable-adjusted effect estimates are used in the MR analysis (*12*).

## RESULTS

**Fig. 1.**
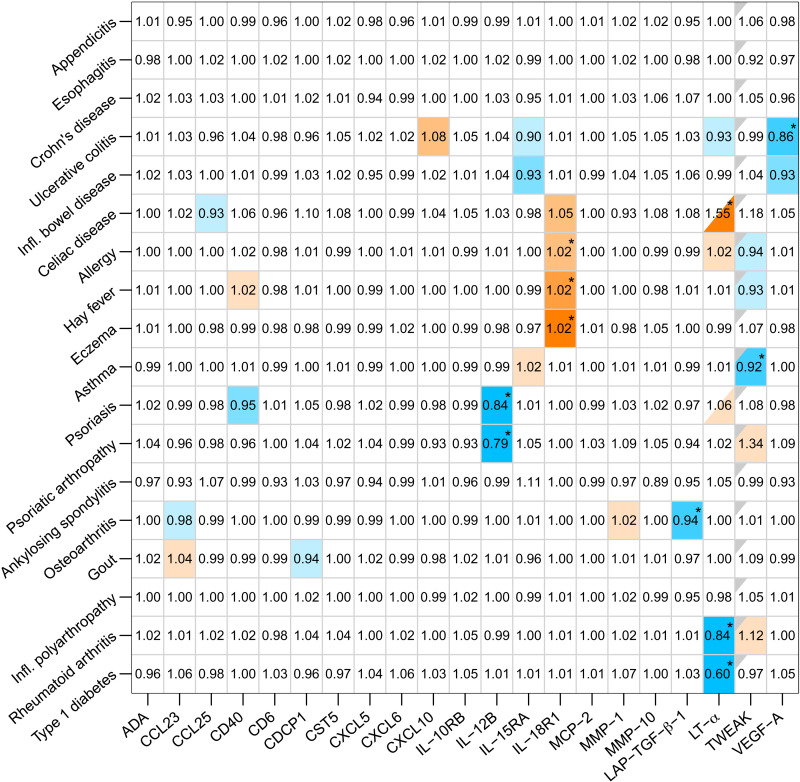
MR results for each biomarker on each inflammatory disease. Main results for the GSMR analysis with *P* value threshold of *P* = 10^−6^ for instrument selection and pairwise LD between instruments of *R*^2^ = 0.6. The values represent ORs that denote the change in disease odds for one SD increase in rank-transformed (INT) biomarker level. An asterisk (*) indicates a significant effect after adjusting for multiple testing (FDR < 0.05), which corresponds to a raw *P* value of <1.4 × 10^−3^ in the main analysis. The color coding is as follows: blue, protective effect; orange, harmful effect; white, nonsignificant result (*P* ≥ 0.05). Unadjusted (raw) *P* values below 0.05 are divided into four different categories and color-coded such that a darker shade denotes a lower *P* value: 0.01 ≤ *P* < 0.05 (light), 0.001 ≤ *P* < 0.01 (medium light), 0.0001 ≤ *P* < 0.001 (medium dark), and *P* < 0.0001 (dark). Half-colored squares denote estimates with *P* < 0.05, similar to full-colored squares, but due to the removal of outlier single-nucleotide polymorphisms (SNPs), the results are based on less than four instruments, which makes the estimates more uncertain. For TWEAK, only four SNPs met the initial requirements, wherefore the HEIDI-outlier removal procedure was switched off. Corresponding results are indicated by squares with gray upper-left corners.

Eighteen inflammatory diseases (Table 1) were identified to have at least 1000 cases in UKB (table S1) and were selected for downstream analysis, and as many as 21 proteins fulfilled the criteria of having at least four instruments *(P* < 10^−6^), with pairwise linkage disequilibrium (LD) lower than *R*^2^ = 0.6 (table S2). The largest number of identified instruments for a single protein was 93, for the interleukin-18 receptor (IL-18R1, encoded by *IL18R1*). The variance explained by a single instrument varied from 2 to 42%, and the total amount of variance explained by all instruments for a single protein ranged from 12 to 60% (table S2). In our main analyses, biomarkers were analyzed in relation to the 18 inflammatory diseases using the Generalized Summary data–based Mendelian Randomization method (GSMR), which incorporates the Heterogeneity-in-Dependent-Instruments (HEIDI)–outlier removal procedure (*13*). In total, 11 significant effects with a false discovery rate (FDR) below 0.05 were identified in the main analysis (Fig. 1 and Table 2). Among those, six biomarkers—IL-12 subunit β (IL-12B; encoded by *IL12B*), IL-18R1, lymphotoxin-α (LT-α; encoded by *LTA*), previously known as tumor necrosis factor–β (TNF-β), TNF ligand superfamily member 12 (TWEAK, encoded by *TNFSF12*), vascular endothelial growth factor A (VEGF-A; encoded by *VEGFA*), and transforming growth factor–β-1 proprotein (LAP-TGF-β-1; encoded by *TGFB1*)—showed effects on one or more diseases (Table 2). For IL-12B, we found protective effects against psoriasis [odds ratio (OR) = 0.84, 95% confidence interval (CI) = 0.80 to 0.88; fig. S1] and psoriatic arthropathy (OR = 0.79, 95% CI = 0.72 to 0.87; fig. S2). LT-α showed protective effects against type 1 diabetes (OR = 0.60, 95% CI = 0.51 to 0.70; fig. S3) and rheumatoid arthritis (OR = 0.84, 95% CI = 0.78 to 0.90; fig. S4). TWEAK showed a protective effect against asthma (OR = 0.92, 95% CI = 0.88 to 0.97; fig. S5), VEGF-A showed a protective effect against ulcerative colitis (OR = 0.86, 95% CI = 0.79 to 0.94; fig. S6), and LAP-TGF-β-1 showed a protective effect against osteoarthritis (OR = 0.94, 95% CI = 0.91 to 0.98; fig. S7). IL-18R1 showed harmful effects on several diseases: hay fever (OR = 1.02, 95% CI = 1.01 to 1.03; fig. S8), allergy (OR = 1.02, 95% CI = 1.01 to 1.03; fig. S9), and eczema (OR = 1.02, 95% CI = 1.01 to 1.03; fig. S10). In addition, we observed a harmful effect of LT-α on celiac disease (OR = 1.55, 95% CI = 1.34 to 1.79; fig. S11). However, the causal effect estimates of LT-α on celiac disease might be biased and should be interpreted with care. This estimate is only based on two instruments, of which one of the instruments, rs2229092, is a missense variant in *LTA*, not annotated to be deleterious. It is therefore possible that the variant influences antibody affinity rather than LT-α levels and that it is invalid as an instrument. In contrast to celiac disease, rs2229092 was removed as a pleiotropic outlier in both analyses for type 1 diabetes and for rheumatoid arthritis.

**Table 1.** Baseline characteristics for participants in NSPHS and in UKB included in the study.

**Baseline** **characteristics**	**NSPHS**	**UKB**
Number of participants	872	361,977
Females, *N* (%)	457 (52.4)	194,809 (53.8)
Age, median (1st–3rd quartile)	50 (34–67)	58 (51–63)
**Inflammatory** **diseases**	**UKB cases**	UKB controls*
Appendicitis	3,798	358,179
Esophagitis	8,984	352,993
Crohn’s disease	3,424	358,553
Ulcerative colitis	1,771	360,206
Inflammatory bowel disease	4,898	357,079
Celiac disease	1,616	360,361
Allergy	27,826	334,151
Hay fever	23,229	338,748
Eczema	12,942	349,035
Asthma	47,628	314,349
Psoriasis	5,542	356,435
Psoriatic arthropathy	1,025	360,952
Ankylosing spondylitis	1,265	360,712
Osteoarthritis	58,587	303,390
Gout	6,830	355,147
Inflammatory polyarthropathy	16,302	345,675
Rheumatoid arthritis	6,097	355,880
Type 1 diabetes	2,577	359,400

*In UKB, all participants that were not identified as being cases for respective disease were used as controls.

**Table 2.** Main results from the MR study, including sensitivity analyses with alternative methods.

**Method**			**GSMR (main analysis)**		**Inverse-variance** **weighted**		**Weighted median**		**MR-Egger**		**MR-Egger** intercept*
**Biomarker**	**Disease**	**No. of IVs** **GSMR/** **other^†^**	**OR (95% CI)**	***P* ^‡^**	**OR** **(95% CI)**	***P* ^‡^**	**OR** **(95% CI)**	***P* ^‡^**	**OR** **(95% CI)**	***P* ^‡^**	***P***
IL-12B	Psoriatic arthropathy	19/22	0.79 (0.72–0.87)	4.5 × 10^−6^	0.81 (0.69–0.96)	1.5 × 10^−2^	0.72 (0.65–0.80)	2.7 × 10^−10^	0.86 (0.73–1.01)	6.5 × 10^−2^	3.5 × 10^−1^
	Psoriasis	16/22	0.84 (0.80–0.88)	2.7 × 10^−13^	0.86 (0.77–0.97)	1.3 × 10^−2^	0.82 (0.78–0.86)	1.2 × 10^−13^	0.87 (0.79–0.95)	3.1 × 10^−3^	8.1 × 10^−1^
IL-18R1	Eczema	93/93	1.02 (1.01–1.03)	7.0 × 10^−5^	1.02 (1.01–1.04)	1.6 × 10^−4^	1.05 (1.04–1.06)	1.1 × 10^−17^	1.02 (1.01–1.04)	1.5 × 10^−4^	5.8 × 10^−1^
	Hay fever	74/93	1.02 (1.01–1.03)	5.3 × 10^−4^	1.01 (0.99–1.02)	2.6 × 10^−1^	1.02 (1.00–1.03)	5.8 × 10^−3^	1.01 (0.99–1.02)	3.3 × 10^−1^	5.1 × 10^−1^
	Allergy	81/93	1.02 (1.01–1.02)	1.4 × 10^−3^	1.01 (1.00–1.02)	1.2 × 10^−1^	1.01 (1.00–1.02)	5.8 × 10^−3^	1.01 (1.00–1.02)	8.5 × 10^−2^	8.9 × 10^−1^
LAP-TGF-β-1	Osteoarthritis	5/5	0.94 (0.91–0.98)	9.0 × 10^−4^	0.94 (0.91–0.98)	1.2 × 10^−3^	0.95 (0.92–0.98)	1.6 × 10^−3^	0.91 (0.84–0.99)	2.5 × 10^−2^	3.5 × 10^−1^
LT-α	Type 1 diabetes	4/6	0.60 (0.51–0.70)	2.4 × 10^−10^	1.01 (0.73–1.40)	9.4 × 10^−1^	1.02 (0.92–1.13)	7.2 × 10^−1^	1.21 (1.07–1.36)	2.3 × 10^−3^	7.9 × 10^−9^
	Rheumatoid arthritis	5/6	0.84 (0.78–0.90)	4.1 × 10^−6^	0.99 (0.89–1.11)	9.2 × 10^−1^	0.96 (0.91–1.01)	1.2 × 10^−1^	1.06 (1.01–1.12)	2.5 × 10^−2^	7.4 × 10^−7^
	Celiac disease	2/6	1.55 (1.34–1.79)	5.7 × 10^−9^	1.14 (0.46–2.81)	7.8 × 10^−1^	1.51 (1.33–1.72)	1.2 × 10^−10^	1.31 (0.91–1.88)	1.4 × 10^−1^	1.5 × 10^−1^
TWEAK	Asthma	4^§^/4	0.92 (0.88–0.97)	6.0 × 10^−4^	0.92 (0.88–0.97)	2.1 × 10^−3^	0.93 (0.90–0.97)	8.2 × 10^−5^	0.82 (0.68–1.00)	4.8 × 10^−2^	2.2 × 10^−1^
VEGF-A	Ulcerative colitis	5/5	0.86 (0.79–0.94)	8.7 × 10^−4^	0.86 (0.79–0.94)	8.2 × 10^−4^	0.85 (0.79–0.92)	5.4 × 10^−5^	0.74 (0.50–1.09)	1.3 × 10^−1^	4.0 × 10^−1^

*The MR-Egger intercept tests whether there is any directional pleiotropy in the data. For *P* values above 0.05, the null hypothesis of no directional pleiotropy is accepted.

†The number of genetic instrumental variables (IVs) used in the MR estimation, where the first number denotes the final number of SNPs used in GSMR after pleiotropic outliers have been removed by the HEIDI-outlier removal procedure, while the second number denotes the original number of SNPs (no removal) used in the three alternative methods in the sensitivity analyses.

‡Raw (unadjusted) *P* values are given. All results from the main GSMR analysis presented in this table reached significance after adjusting for multiple testing (FDR < 0.05). See Table 3 for corresponding adjusted *P* values (FDR) in the GSMR analysis.

§For TWEAK, only four SNPs met the initial requirements and the HEIDI-outlier procedure in GSMR was therefore switched off.

We applied several alternative MR methods as part of the sensitivity analyses, including the inverse-variance weighted, weighted median, and the MR-Egger methods. These results were consistent with the main analysis, i.e., they showed the same direction of effect (Table 2 and figs. S1 to S11), even if not all effects were significant in all sensitivity analyses. The only contradictive effects were those of LT-α on rheumatoid arthritis and on type 1 diabetes with the MR-Egger method. However, in the sensitivity analyses, the MR-Egger intercept deviated significantly from zero (Table 2), suggesting that the analyses may be affected by pleiotropy, which agrees with the GSMR analysis where several LT-α instruments were flagged as outliers and removed before effect estimation (figs. S3 and S4). Recall that no outliers were removed in any of the methods used for the sensitivity analyses (Table 2).

We also performed sensitivity analyses, where more conservative parameter settings were used in GSMR (Table 3 and figs. S12 to S14). This resulted in fewer instruments identified, and not all biomarkers could therefore be analyzed, or they had too few (<5) instruments for the HEIDI-outlier analysis to work properly. Apart from LT-α, all results from the main analysis replicated (FDR < 0.05) in all settings where the analysis was feasible, with the one exception being the effect of IL-18R1 on hay fever, which was only nominally significant in one of the stricter settings (Table 3). Significant relationships (FDR < 0.05), in addition to those of the main analysis, were found for some of the stricter settings (Table 3), such as a protective effect of CD40 on psoriasis (OR = 0.92, 95% CI = 0.88 to 0.96) and harmful effects of IL-18R1 on asthma (OR = 1.02, 95% CI = 1.01 to 1.02 and OR = 1.02, 95% CI = 1.01 to 1.03) and on celiac disease (OR = 1.06, 95% CI = 1.02 to 1.09). For LT-α, which was highlighted in the main analyses to have one or several invalid instruments, the HEIDI-outlier procedure was disabled in these sensitivity analyses because of too few remaining instruments. It is therefore not unexpected that the causal estimates for LT-α in these sensitivity analyses partly disagree with the main analyses (Table 3). We also identified an additional protective effect of LT-α on psoriasis (OR = 0.83, 95% CI = 0.77 to 0.89), but due to suspected pleiotropy, this result is potentially biased. In general, we advise against an overinterpretation of any results for LT-α in which rs2229092 and other invalid instruments are included in the causal effect estimation.

**Table 3.** Results from the GSMR analysis with more stringent thresholds for the SNP correlations (*R*^2^) and SNP significance levels (*P*). Causal effect estimates with FDR < 0.05 in any of the sensitivity analyses with stricter parameter settings are included.

**Thresholds**		***R*^2^ = 0.6; *P* = 1 × 10^−6^ (main analysis)**				***R*^2^ = 0.2; *P* = 1 × 10^−6^**				***R*^2^ = 0.6; *P* = 5 × 10^−8^**				***R*^2^ = 0.2; *P* = 5 × 10^−8^**			
**Biomarker**	**Disease**	**No. of** IVs*	**OR** **(95% CI)**	***P***	**FDR^†^**	**No. of** IVs*	**OR** **(95% CI)**	***P***	**FDR^†^**	**No. of** IVs*	**OR** **(95% CI)**	***P***	**FDR^†^**	**No. of** IVs*	**OR** **(95% CI)**	***P***	**FDR^†^**
CD40	Psoriasis	22	0.95 (0.92–0.99)	6.6 × 10^−3^	1.8 × 10^−1^	11	0.98 (0.93–1.02)	3.1 × 10^−1^	8.6 × 10^−1^	15	0.92 (0.88–0.96)	2.3 × 10^−4^	1.9 × 10^−2^	9	0.96 (0.92–1.01)	1.1 × 10^−1^	8.4 × 10^−1^
IL-12B	Psoriatic arthropathy	19	0.79 (0.72–0.87)	4.5 × 10^−6^	3.4 × 10^−4^	15	0.82 (0.74–0.91)	2.3 × 10^−4^	1.4 × 10^−2^	10	0.73 (0.64–0.83)	1.4 × 10^−6^	2.2 × 10^−4^	8	0.63 (0.54–0.73)	5.2 × 10^−10^	1.7 × 10^−7^
	Psoriasis	16	0.84 (0.80–0.88)	2.7 × 10^−13^	1.0 × 10^−10^	13	0.89 (0.84–0.93)	5.1 × 10^−7^	9.6 × 10^−5^	8	0.82 (0.77–0.87)	1.1 × 10^−11^	3.6 × 10^−9^	6	0.86 (0.81–0.92)	1.0 × 10^−5^	1.6 × 10^−3^
IL-18R1	Asthma	78	1.01 (1.00–1.01)	1.6 × 10^−1^	7.5 × 10^−1^	47	1.02 (1.01–1.02)	5.0 × 10^−4^	2.0 × 10^−2^	48	1.01 (1.00–1.02)	4.0 × 10^−2^	5.7 × 10^−1^	35	1.02 (1.01–1.03)	1.1 × 10^−4^	7.1 × 10^−3^
	Eczema	93	1.02 (1.01–1.03)	7.0 × 10^−5^	4.4 × 10^−3^	49	1.03 (1.01–1.05)	1.9 × 10^−4^	1.4 × 10^−2^	63	1.03 (1.01–1.04)	2.6 × 10^−5^	2.8 × 10^−3^	38	1.04 (1.02–1.06)	1.5 × 10^−5^	1.6 × 10^−3^
	Hay fever	74	1.02 (1.01–1.03)	5.3 × 10^−4^	2.8 × 10^−2^	43	1.03 (1.01–1.04)	3.1 × 10^−5^	3.0 × 10^−3^	49	1.01 (1.00–1.02)	4.2 × 10^−2^	5.7 × 10^−1^	31	1.03 (1.02–1.05)	2.4 × 10^−5^	1.9 × 10^−3^
	Allergy	81	1.02 (1.01–1.02)	1.4 × 10^−3^	4.8 × 10^−2^	45	1.02 (1.01–1.03)	5.2 × 10^−4^	2.0 × 10^−2^	52	1.02 (1.01–1.03)	7.7 × 10^−4^	4.0 × 10^−2^	33	1.02 (1.01–1.04)	2.7 × 10^−4^	1.5 × 10^−2^
	Celiac disease	92	1.05 (1.02–1.08)	3.2 × 10^−3^	1.0 × 10^−1^	49	1.05 (1.01–1.10)	2.3 × 10^−2^	4.1 × 10^−1^	63	1.06 (1.02–1.09)	6.4 × 10^−4^	4.0 × 10^−2^	38	1.05 (1.00–1.10)	6.6 × 10^−2^	8.2 × 10^−1^
LAP-TGF-β-1	Osteoarthritis	5	0.94 (0.91–0.98)	9.0 × 10^−4^	3.4 × 10^−2^	3^‡^	0.94 (0.90–0.97)	3.6 × 10^−4^	1.9 × 10^−2^	1^‡^	NA^§^	NA^§^	NA^§^	1^‡^	NA^§^	NA^§^	NA^§^
LT-α^║^	Type 1 diabetes	4	0.60 (0.51–0.70)	2.4 × 10^−10^	4.5 × 10^−8^	4^‡^	0.94 (0.87–1.02)	1.5 × 10^−1^	7.3 × 10^−1^	3^‡^	0.99 (0.90–1.08)	7.9 × 10^−1^	9.6 × 10^−1^	2^‡^	0.93 (0.85–1.03)	1.5 × 10^−1^	8.4 × 10^−1^
	Rheumatoid arthritis	5	0.84 (0.78–0.90)	4.1 × 10^−6^	3.4 × 10^−4^	4^‡^	0.97 (0.92–1.01)	1.8 × 10^−1^	7.4 × 10^−1^	3^‡^	1.00 (0.95–1.05)	9.9 × 10^−1^	1.0 × 10^−0^	2^‡^	0.98 (0.93–1.03)	3.8 × 10^−1^	9.1 × 10^−1^
	Psoriasis	2	1.06 (1.01–1.11)	1.5 × 10^−2^	2.9 × 10^−1^	4^‡^	0.83 (0.77-0.89)	3.3 × 10^−7^	9.6 × 10^−5^	3^‡^	1.00 (0.96–1.05)	9.2 × 10^−1^	1.0 × 10^−0^	2^‡^	1.06 (1.01–1.11)	1.5 × 10^−2^	4.0 × 10^−1^
	Celiac disease	2	1.55 (1.34–1.79)	5.7 × 10^−9^	7.1 × 10^−7^	4^‡^	1.33 (1.18–1.49)	2.7 × 10^−6^	3.4 × 10^−4^	3^‡^	0.98 (0.87–1.10)	7.3 × 10^−1^	9.3 × 10^−1^	2^‡^	1.15 (1.01–1.31)	3.0 × 10^−2^	5.6 × 10^−1^
TWEAK	Asthma	4^‡^	0.92 (0.88–0.97)	6.0 × 10^−4^	2.8 × 10^−2^	2^‡^	0.92 (0.88–0.96)	4.9 × 10^−4^	2.0 × 10^−2^	1^‡^	NA^§^	NA^§^	NA^§^	1^‡^	NA^§^	NA^§^	NA^§^
VEGF-A	Ulcerative colitis	5	0.86 (0.79–0.94)	8.7 × 10^−4^	3.4 × 10^−2^	2^‡^	0.85 (0.78–0.93)	7.5 × 10^−4^	2.6 × 10^−2^	5	0.86 (0.79–0.94)	8.7 × 10^−4^	4.0 × 10^−2^	2^‡^	0.85 (0.78–0.93)	7.5 × 10^−4^	3.5 × 10^−2^

*The number of IVs that remained after HEIDI-outliers had been removed.

†*P* value adjusted for multiple testing (FDR). Note that the region of raw *P* values corresponding to FDR < 0.05 differs slightly between the four different analyses.

‡After nonsignificant and/or correlated SNPs were removed, the number of remaining SNPs was too few (<5) for the HEIDI-outlier procedure to work properly, wherefore HEIDI was switched off in these analyses.

§After nonsignificant and/or correlated SNPs were removed, only a single SNP remained, wherefore GSMR could not estimate a causal effect.

║As highlighted in the main analyses, some of the instruments for LT-α might be invalid. In these sensitivity analyses, the HEIDI-outlier procedure had to be switched off due to the low number of instruments, and the potentially invalid instruments are therefore included in the causal estimation, in particular, rs2229092.

## DISCUSSION

In this study, we performed a two-sample MR analysis and found 6 of 21 inflammatory protein biomarkers to be causally involved in inflammatory disease development. A biomarker is commonly found to be more abundant in patients with disease. However, our results indicate that a genetic predisposition to higher levels of the identified biomarkers is often protective against disease development. The only exception was that of IL-18R1, where high protein levels cause increased risk of allergy, hay fever, and eczema. IL-18R1 is a receptor that is essential for IL-18–mediated signal transduction through the binding of IL-18 to both IL-18RAP (IL-18R accessory protein) and IL-18R1 (*14*). IL-18 activity has previously been linked to several autoimmune diseases, and preventing IL-18 from binding to IL-18R1 reduces the risk of inflammatory diseases (*14*). Higher levels of IL-18R1 could instead increase the activity of the IL-18–mediated signal, which agrees with our results. We therefore propose a causal mechanism, where IL-18 signaling is promoted through the increased expression of the IL-18R1 receptor (*14*). Harmful effects of IL-18R1 on asthma and celiac disease were also identified, but only in some sensitivity analyses where we adopted stricter thresholds for the genetic instruments. This shows that selection of instruments for MR analyses can potentially influence downstream results.

To our knowledge, this is the first study to show a protective effect of LT-α on both type 1 diabetes and rheumatoid arthritis. LT-α is a cytokine in the TNF superfamily of signaling molecules and receptors. It binds to TNFR1 and TNFR2 (TNF receptors 1 and 2), which are the primary receptors for TNF-α (also called TNF) (*15*). TNF-α has previously been shown to be involved in the development of both rheumatoid arthritis (*16*) and type 1 diabetes (*17*). TNF signaling is a therapeutic target for inflammatory disease, and more than 20 agents that target the TNF-signaling pathway are currently in clinical use (*18*, *19*). Drugs that inhibit TNF have also been shown to reduce inflammation in rheumatic arthritis patients (*20*) and are commonly used in the clinic. Previous studies have presumed that high levels of LT-α also increase risk of inflammatory diseases, primarily due to its sequence similarity with TNF-α (*16*). However, receptor affinity for LT-α is just slightly lower compared to TNF-α, while LT-α has been shown to be 100- to 1000-fold less potent in the receptor activating (*15*). Higher genetic levels of LT-α could therefore compete with TNF-α in binding to TNFR1 and TNFR2 and thereby act as an antagonist to TNF-α, which agrees with the protective effect identified in our study.

For IL-12B, we found a protective effect of elevated biomarker levels against developing psoriasis and psoriatic arthropathy. However, reduction of IL-12B signaling is the therapeutic aim of the IL-12B–targeting monoclonal antibody ustekinumab, which is used to treat symptoms of both psoriasis and psoriatic arthropathy. Even if this appears to contradict our results, similar effects have been seen in a previous MR study (*6*). IL-12B is a subunit to both the heterodimeric cytokines IL-12 and IL-23 (*21*), which are both important for host defense against pathogens and wound healing (*22*). Our results suggest that the beneficial effects of these cytokines in protecting healthy tissue from damage could be very different from the effects of an excess amount of cytokines produced in response to the chronic inflammatory state that characterize psoriasis and psoriatic arthropathy.

We found that VEGF-A protects against ulcerative colitis, which is one subtype of inflammatory bowel disease. A previous study has identified an overexpression of VEGF-A in mice with colitis and in patients with inflammatory bowel disease (*23*). It is known that VEGF-A expression is increased in oxygen-deprived cells (*24*), and hypoxia is a well-known characteristics of inflammation (*25*) and colitis (*26*). It is therefore not surprising that VEGF-A is expressed in response to hypoxia in patients with inflammatory bowel disease, as an indicator of mucosal inflammation (*26*). VEGF-A is an important factor in the formation of new blood vessels, which is essential for wound healing. In cancer, anti-VEGF therapy using bevacizumab is used to reduce the growth of new blood vessels (*27*). However, bowel perforation is a serious side effect of bevacizumab because the intestinal mucosa may be susceptible to ulcers and perforation as a result of the VEGF inhibition (*28*). This highlights the importance of VEGF-A in healthy bowel mucosa and supports our findings that VEGF-A protects against developing ulcerative colitis. In contrast to this finding, no causal effect of VEGF-A on Crohn’s disease was detected, despite almost twice the number of cases of Crohn’s disease compared to ulcerative colitis in UKB. These two types of inflammatory bowel disease partly differ in the location of where they occur in the intestines, and the contrasting results in our analyses may therefore suggest molecular differences between the two subtypes.

We further showed that higher LAP-TGF-β-1 protects against developing osteoarthritis. LAP-TGF-β-1 is the TGF-β-1 proprotein, which is split into LAP and TGF-β-1. In humans, there are three homologs of the TGF-β where TGF-β-1 is the one predominantly expressed in the articular cartilage, the tissue around the bones in the joints (*29*). It has been suggested that TGF-β plays a critical role in the development of osteoarthritis (*30*, *31*). In healthy joints, active TGF-β is normally expressed only for a short period of time after joint loading. However, in patients with osteoarthritis, constant high levels of TGF-β have been observed (*31*), although this is more likely to be a consequence of the disease. Our result is also supported by a recent clinical trial that demonstrated beneficial effects with allogeneic cell therapy, where chondrocytes that were transduced to express TGF-β-1 were injected into osteoarthritic joints (*32*).

We also showed that TWEAK protects against asthma. A recent study showed that TWEAK levels are increased in children with asthma, and that TWEAK levels positively correlate with degree of airway obstruction (*33*). After acute injury, TWEAK coordinates tissue inflammation and tissue regeneration to reestablish normal tissue (*34*). However, a constant up-regulation of TWEAK might instead have pathogenic effects and cause tissue damage, and the focus has therefore been on inhibiting TWEAK to cure symptoms of disease that have already developed (*35*). The protective effect of higher TWEAK levels that we find in our study may hypothetically be due to the protective effects against tissue damage in healthy individuals, which would result in a risk reduction of developing asthma. The TWEAK-Fn14 signaling axis has also been under investigation in clinical trials but have yet to progress past phase 1 trials (*36*, *37*).

It is not surprising that causal effects were not observed for all proteins in our study even if the proteins were selected to be biomarkers of disease and the abundance of many of the proteins have previously been linked to inflammatory diseases or processes. Increased protein levels may instead represent a natural response to disease, rather than a causal factor that leads to disease. The predominant direction of causality between disease and disease-related protein biomarkers could be unraveled by performing a bidirectional MR analysis. However, we did not have the power to investigate the effect of disease status on biomarker levels, and we are therefore unable to draw any conclusions about the strength of the reversed causal effect, which is a limitation of the current study. It is also possible that we were unable to identify strong enough instruments for some proteins, which would decrease our ability to identify causal effects. However, the relatively large variance explained by the genetic instruments for most proteins speaks against this possibility. It appears instead that the effects of most proteins on disease risk are truly absent or at least too small to have any clinical relevance. Proteins with such small effects may arguably be less useful as drug targets. We note though that the causal estimates presented here should, on average, be slightly conservative, partly because any weak-instrument bias goes in the direction of the null in a two-sample MR (*38*) and partly because any winner’s curse bias introduced by using the same cohort (NSPHS) for both instrument identification and effect estimation also goes in the direction of the null in a two-sample MR (*38*). This bias may decrease the power to detect causal effects (*39*). To compensate for this potential loss of power, we adopted a more liberal LD threshold to increase the number of genetic instruments per protein (*40*). Ideally, independent or nearly independent single-nucleotide polymorphisms (SNPs) are used as instruments in a two-sample MR. However, because the expression levels of a protein is more monogenetic compared to most complex phenotypes, it is challenging to find completely independent instruments. The main MR approach used in this study (GSMR) and several of the alternative methods used in the sensitivity analyses (inverse-variance weighted and MR-Egger) are all able to account for correlated SNPs via the variance-covariance matrix. We could therefore select SNPs that are partly in LD without introducing bias in the estimates of the standard errors. As supported by the sensitivity analysis presented in Table 3, the main results are also generally robust to changes in LD threshold, which suggests that the LD pattern is adequately accounted for in the main analysis.

Disease diagnoses in UKB are self-reported or from registry data, rather than being validated by a specialist directly from the medical record or in the clinic. This might lead to a recall bias and misclassification. However, considering the large number of disease cases discussed here (*N* > 1000), a small number of misdiagnosed cases should not likely influence the results markedly. Disease diagnoses were validated by the UKB team before releasing the data for our study (*41*). One should, however, be aware of the fact that any misclassification of a disease condition could lead to reduced power in our study and an underestimation of the true causal effect of the biomarkers on disease risk. Another limitation with UKB is that only 6% of the individuals that were invited to UKB volunteered to participate, and the invitees represent those who survived long enough to be able to participate. This have presumably led to survival bias toward a healthier population within the UKB cohort, compared to the population as a whole, although inflammatory disorders might be less affected as they often are not directly lethal to the individual. Also, a recent study compared risk factor–disease estimations in UKB with those from 18 nationally representative studies with conventional response rates. Their results showed that studies like UKB do have a scientific value despite low response rates (*42*). The study concluded that despite more favorable levels of baseline characteristics and disease-specific mortality in UKB, etiological findings from UKB are generalizable to England and Scotland.

In summary, we identified six proteins that have causal effects on 11 of the inflammatory diseases investigated. Our results suggest that many proteins are expressed to protect against damage, rather than being causal for disease development, and these proteins are likely to be overexpressed in diseased individuals. This trend is further supported by our sensitivity analyses, where a significant protective effect of CD40 on risk of developing psoriasis was also found. Both CD40 and the CD40 ligand have previously been shown to be highly expressed in psoriatic lesions (*43*). They are also up-regulated in psoriatic patients, which indicate their involvement in the pathogenesis of psoriasis (*44*). However, the causal effects on disease symptoms were not investigated, and it is unclear whether the identified proteins have the same action in healthy and diseased individuals. Our results not only confirm previous findings from model organisms but also highlight several established and experimental drug targets that have already entered clinical trials. The design of our study could therefore serve as a guideline for a more efficient evaluation of potential drug targets.

## MATERIALS AND METHODS

### The NSPHS

NSPHS was a health survey to investigate medical consequences of lifestyle and genetics. A total of 719 individuals from Karesuando and 350 individuals from Soppero county of Norrbotten, Sweden were recruited in 2006 and 2009 (Table 1). For each participant, blood samples were taken and serum and plasma were separated and immediately frozen and stored at −70°C (*2*). The NSPHS was approved by the ethics committee at Uppsala University (Regionala Etikprövningsnämnden, Uppsala, 2005:325), and the approval was extended on 9 March 2016, in compliance with the Declaration of Helsinki.

The protein levels for 92 putative biomarkers of inflammation had been measured using the Olink Proseek Multiplex Inflammation panel (INF v.3001, www.olink.com) and the protein extension assay, as previously described (*11*, *45*). Briefly, an affinity-based assay is used with pairs of oligonucleotide-labeled antibody probes that bind to each of the targeted proteins. If both antibodies bind in close proximity, a polymerase chain reaction (PCR) target is produced and quantified using standard real-time PCR. The samples were analyzed on 10 different plates with 96 wells each (92 for samples, 1 for a negative control, and 3 for positive controls). The controls are used to determine the lower detection limit and to normalize the measurements. Measurements below the detection limit were removed from further analysis. In total, 903 samples were analyzed, of which 892 passed the protein initial quality control (QC) that has been described previously (*1*, *2*, *45*). Protein measurements were adjusted for their position on the plate and standardized using a conservative method where the protein levels for each protein were transformed using the rank-based inverse normal transformation (INT), with mean = 0 and SD = 1, within each plate. To achieve enough power for downstream analyses, only proteins that were above the detection level in at least 400 (46%) of the samples with WGS data were included. Eighty-five of 92 inflammatory proteins measured passed QC.

A total of 1041 samples from NSPHS have been sequenced using Illumina short read technology (X-ten) to 30× coverage per individual. Preparation, sequencing, variant calling, and QC have been described previously (*46*). Briefly, WGS data were aligned to the hg19 (GRCh37) reference genome using bwa-mem. Raw alignments were processed according to the Genome Analysis Toolkit (GATK) best practice, and variants were then called with GATK HaplotypeCaller 3.3. Genetic outliers, potentially contaminated samples, and individuals with sex discordance errors were removed during QC, which left 1021 unique samples with WGS data and a total of 16,890,549 variants called. In the GWAS, only biallelic SNPs were included, which left 12,210,410 genetic variants. A minor allele frequency (MAF) cutoff of 0.0017 (minor allele count of 3) and a *P* value cutoff for Hardy-Weinberg equilibrium of 5 × 10^−8^ were used in the GWAS for the proteins. However, only SNPs that overlapped with the filtered UKB SNP set (see the next section) were considered when instruments for the MR were selected. All GWASs were performed using the R package GenABEL (*47*, *48*) as previously described (*11*), with the rank-transformed (INT) protein levels as response, while adjusting for age, sex, and batch effect. Relatedness was adjusted for using a kinship matrix estimated from 300,000 tagSNPs (*11*). The variance explained by each genetic instrument (SNP) separately and the variances explained by all SNPs jointly for each protein were estimated using the anova.glm function in R. In total, 872 participants passed both biomarker and SNP QC and were included in the biomarker GWASs.

### UK Biobank

The UKB includes 502,682 participants from all across the United Kingdom, aged 37 to 73 years at the recruitment between 2006 and 2010. The UKB study was approved by the National Research Ethics Committee (REC reference 11/NW/0382). Informed consent to the study was given by all participants. Use of UKB data has been approved by UKB (application nr: 8260). The UKB analysis performed in this study has also been approved by the Swedish Ethical Review Authority (dnr: 2020-04415). Health variables, including inflammatory disease status, have been collected through questionnaires, interviews, and hospital records.

A total of 438,417 UKB participants had been genotyped using the UKB Axiom array, and another 49,994 participants had been genotyped using the similar (95% common marker content) UK BiLEVE array. SNPs had been imputed using UK10K and 1000 genomes phase 3, as reference panels (*49*, *50*). For this project, we used imputed data from the third release, which contained a total of 93,093,070 SNPs. After genotype QC, in which variants with MAF < 0.01, *P* values for Hardy-Weinberg equilibrium below 1 × 10^−20^, missing call rate > 0.05, and imputation quality < 0.8 were removed, the remaining UKB SNP set was matched with the set of SNPs that had passed QC in the NSPHS. A total of 8,327,680 SNPs were shared between UKB and NSPHS. These SNPs were included for downstream analyses in both NSPHS and UKB. In UKB, we excluded first- and second-degree relatives using kinship data by using a cutoff for the estimated kinship of 0.044. We also excluded participants with discordance between self-reported and genetic sex, as well as high heterozygosity and more than 5% missing genotypes. Moreover, participants that self-reported not being of white British descent, or who were not classified as Caucasians by principal component analysis, were excluded. This left 361,977 nonrelated participants for downstream analyses.

### Assessment of disease status in UKB

To assess information regarding inflammatory disease status for UKB participants, information was taken from different categories: main and secondary diagnoses made during hospital stay, medical conditions assessed from both verbal interviews and touchscreen questionnaires, and cause of death. For a detailed description of data fields and coding used for each disease and covariate, see table S1. We selected a set of 18 inflammatory diseases, for which at least 1000 participants had a reported diagnosis in UKB, to be analyzed in this study (Table 1).

### Mendelian randomization

In MR, the genetic variant (instrument) must fulfill three fundamental assumptions to be valid (*10*): (i) the variant must be associated with the exposure, (ii) the variant should not be associated with any potential confounder to the exposure—outcome relation, and (iii) the variant should not be directly associated with the outcome, in our case, the disease. Instead, it must only be associated with the disease via the exposure, i.e., unless the causal effect of the exposure on disease is zero. The last two assumptions ensure that the genetic variant is not horizontally pleiotropic. Unfortunately, the validity of assumptions (ii) and (iii) cannot be tested directly for individual instruments, wherefore any causal inference based on a single genetic variant is uncertain and may be marred by large systematic errors. However, the validity may be tested indirectly for a collection of genetic instruments by identifying the potential causal relationship within which valid instruments must fall. Instruments that do not obey this relationship may be classified as pleiotropic outliers (horizontal pleiotropy) and may be removed before the causal effect estimation to avoid bias (*13*, *51*).

To identify instruments for the MR, we first filtered for SNPs that had passed QC in both NSPHS and UKB. Second, we filtered for cis-regulatory SNPs that are located in proximity of the genes that encode the proteins of interest. This was done to minimize the risk of selecting horizontally pleiotropic and invalid instruments. We defined the cis-regulatory window to be between the end points of each protein-coding gene, plus a 2-Mb window on each side. Third, we extracted all SNPs within these windows with a *P* value below a default significance threshold of *P* = 10^−6^ and accepted only SNPs as genetic instruments below a default LD threshold of *R*^2^ = 0.6 in both NSPHS and UKB. This last step was performed in the following way: (i) The SNP with the lowest *P* value within the cis-regulatory window of the protein-coding gene of interest was selected; (ii) all of the associated SNPs in LD (*R*^2^ > 0.6) with the selected SNP, either in NSPHS or in UKB, were removed; (iii) a new SNP, being the one with the lowest *P* value among the remaining SNPs within the window, was selected. The procedure was repeated from step (ii) until no SNP below *P* = 10^−6^ remained. This procedure ensured that none of the genetic instruments are in strong LD (*R*^2^ > 0.6), neither in NSPHS nor in UKB. A *P* value threshold of *P* = 10^−6^ was selected because about 0.1% of the genome was analyzed for each of the 85 proteins, and *P* < 10^−6^ should therefore correspond to about the same level of multiple testing adjustment as used in a GWAS. An LD threshold of *R*^2^ = 0.6 was selected to maintain a meaningful number of genetic instruments for as many proteins as possible and to increase power to detect causal effects (*40*). Only proteins with at least four instruments passing QC and filtering were included in the main MR analysis. A detailed summary of all 343 genetic instruments that were used in this study is provided in table S2.

To assess the potentially causal effects that a protein confers on the risk of developing an inflammatory disease, we performed MR using a two-sample design (*38*). In the first sample (NSPHS), we performed GWAS for the measured proteins and identified IVs. We also used NSPHS to estimate the effects, and corresponding standard errors, of the instruments on protein levels. In the second sample (UKB), we estimated the effects of the instruments on risk of disease with logistic regression, using the glm package in R (version 3.6.1). Sex, age, and the first 20 genetic principal components were included as covariates.

The main MR analyses were performed with the R package gsmr (version 1.0.8), which implements the GSMR method (*13*), to enable causal inference based on multiple SNPs that were tested for horizontal pleiotropy. GSMR incorporates the HEIDI-outlier removal procedure, which identifies and removes instruments that are considered to be statistical outliers before causal effect estimation, because these SNPs are likely to be pleiotropic and thereby invalid as instruments. In the main analysis, the HEIDI-outlier flag in gsmr was set to “TRUE,” except for the biomarker TWEAK, for which the outlier analysis was disabled as there were too few SNPs (<5) for the procedure to work properly. The significance threshold for an SNP being identified as a HEIDI-outlier was set to the default value of α = 0.01. Causal relationships were adjusted for multiple testing using the FDR, for which an FDR below 0.05 was considered significant. Unadjusted, raw *P* values are denoted by *P* in the text.

GSMR uses generalized least-squares to estimate the causal effect (*13*). In this way, both heteroskedastic and correlated errors in the SNP effects on the outcome (disease) can be controlled for. To account for correlated errors in the effect estimates, which originate from the LD pattern between the genetic instruments, the pairwise correlations between the SNPs were estimated in UKB, using all individuals after filtering (*N* = 361,977). This was done to ensure that correlations had the correct sign for all SNPs used in the MR analysis. In GSMR, the estimated standard error is based on a fixed effect model with the test statistic following a χ^2^ distribution with one degree of freedom (*13*).

Apart from the main MR analysis, we also performed two additional sets of sensitivity analyses. In the first set of analyses, we explored the parameter space of the thresholds for significance and LD. A stricter threshold for significance of *P* = 5 × 10^−8^ in the biomarker GWASs (NSPHS), as well as a stricter LD threshold of *R*^2^ = 0.2 between the genetic instruments in UKB, was adopted. Both thresholds were directly tweaked in the gsmr R function by changing the corresponding parameters gwas_thresh and ld_r2_thresh, respectively. In the second set of sensitivity analyses, we applied three additional MR methods to all significant relationships between proteins and diseases from the main analysis. These included the inverse-variance weighted (*52*), weighted median (*53*), and the MR-Egger (*54*) methods, of which the last two are developed to be robust against certain types of horizontal pleiotropy. These analyses were performed by the MendelianRandomization package in R (version 0.4.1). Similar to GSMR, the inverse-variance weighted and the MR-Egger methods accounted for both heteroskedasticity and SNP correlation in the causal estimation via the variance-covariance matrix. However, in contrast to GSMR, the estimated standard error for these two methods is based on a random effect model, with the residual standard error not being allowed to fall below one. The standard error for the weighted median method is estimated by bootstrapping. All instruments were included in this second set of analyses. Outlier SNPs were only removed in those analyses where GSMR was used, unless the HEIDI-outlier procedure was switched off in case there was not enough instruments to allow outlier identification. All MR estimates are reported as ORs per one SD increase in rank-transformed (INT) in protein levels.

## Notes

View/request a protocol for this paper from *Bio-protocol*.
