# Supplementary material for: Causal effects of inflammatory protein biomarkers on inflammatory diseases
Source: Sci Adv. 2021 Dec 8;7(50):eabl4359. doi: 10.1126/sciadv.abl4359 (PMC8654293; doi:10.1126/sciadv.abl4359)
Supplement: Supplementary file 1 — Figs. S1 to S14 Table S1 [file sciadv.abl4359_sm.pdf]

Science Advances
AAAS

Supplementary Materials for

Causal effects of inflammatory protein biomarkers on inflammatory diseases

Weronica E. Ek, Torgny Karlsson, Julia Höglund, Mathias Rask-Andersen, Åsa Johansson*

*Corresponding author. Email: asa.johansson@igp.uu.se

Published 8 December 2021, *Sci. Adv.* **7**, eabl4359 (2021)
DOI: 10.1126/sciadv.abl4359

The PDF file includes:

Figs. S1 to S14
Table S1

Other Supplementary Material for this manuscript includes the following:

Table S2

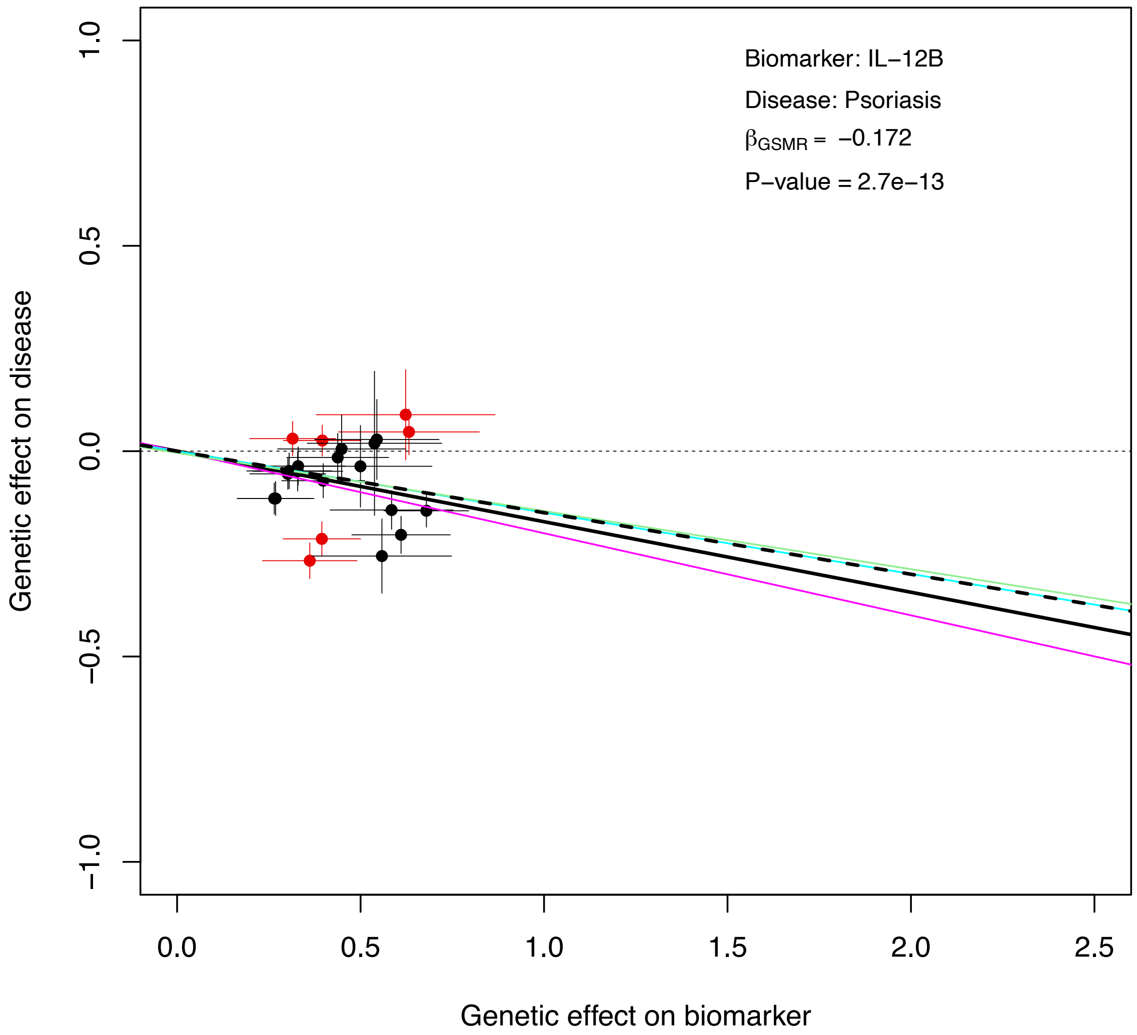

Fig. S1. Causal effect of IL-12B on psoriasis. Genetic instruments are shown with their effects on the biomarker plotted against their effects on disease. Black dots (with corresponding 95% CI) denote SNPs that GSMR identifies as being valid, while red dots (with corresponding 95% CI) are identified as statistical outliers by the HEIDI-outlier procedure. Their effect on disease is likely modified by horizontal pleiotropy and they are therefore removed prior to causal estimation. The black solid line denotes the estimate by GSMR which is adopted as the main result (corresponding raw *P* value is shown in figure legend), while the cyan line denotes the estimate by the inverse variance weighted method, the magenta line denotes the estimate by the weighted median method, and the green line denotes the estimate by the MR-Egger method. Note that in the last three, alternative methods, all genetic variants are used in the causal estimation. Finally, the black dashed line denotes the estimate by GSMR with the HEIDI-outlier removal procedure switched off.

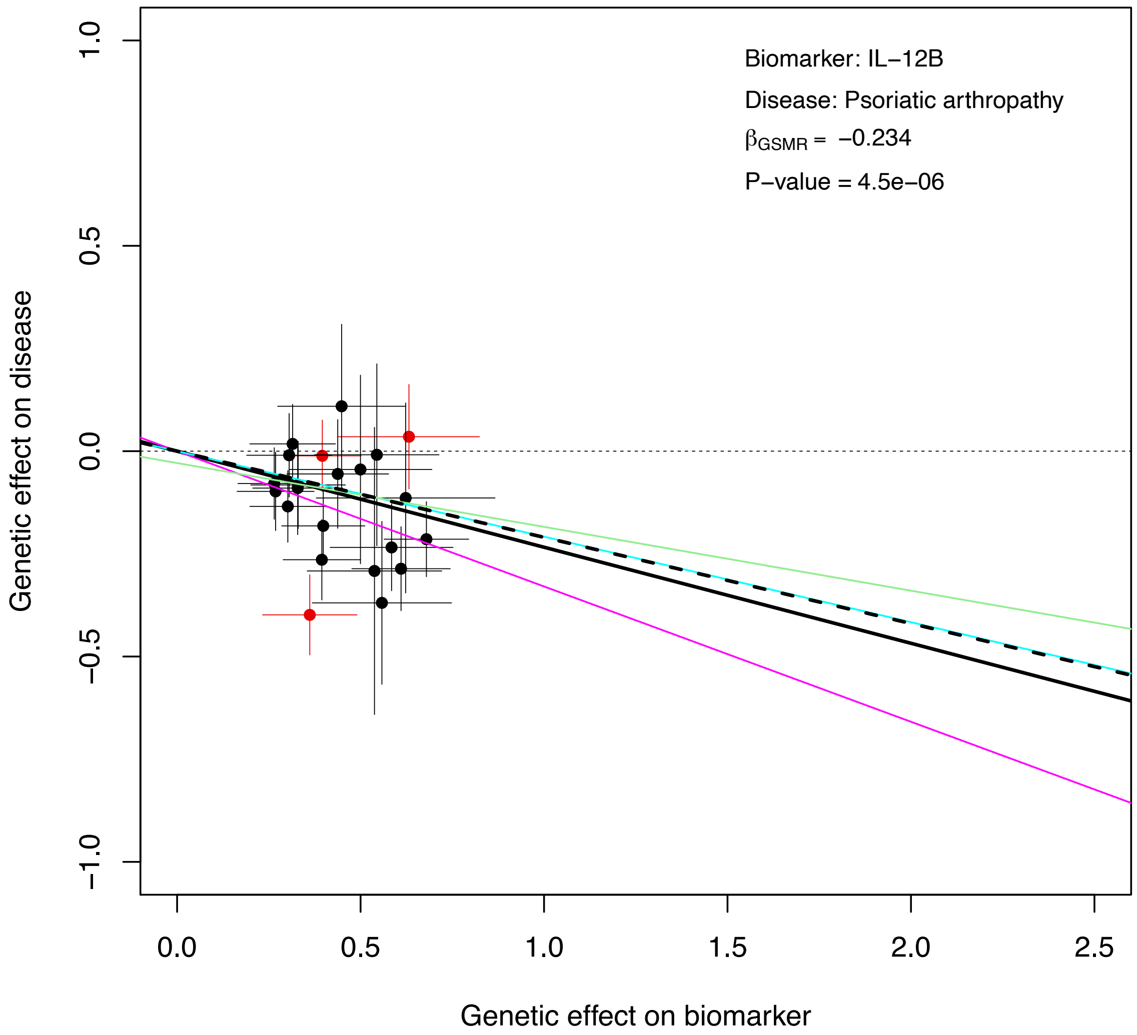

Fig. S2. Causal effect of IL-12B on psoriatic arthropathy. Genetic instruments are shown with their effects on the biomarker plotted against their effects on disease. Black dots (with corresponding 95% CI) denote SNPs that GSMR identifies as being valid, while red dots (with corresponding 95% CI) are identified as statistical outliers by the HEIDI-outlier procedure. Their effect on disease is likely modified by horizontal pleiotropy and they are therefore removed prior to causal estimation. The black solid line denotes the estimate by GSMR which is adopted as the main result (corresponding raw *P* value is shown in figure legend), while the cyan line denotes the estimate by the inverse variance weighted method, the magenta line denotes the estimate by the weighted median method, and the green line denotes the estimate by the MR-Egger method. Note that in the last three, alternative methods, all genetic variants are used in the causal estimation. Finally, the black dashed line denotes the estimate by GSMR with the HEIDI-outlier removal procedure switched off.

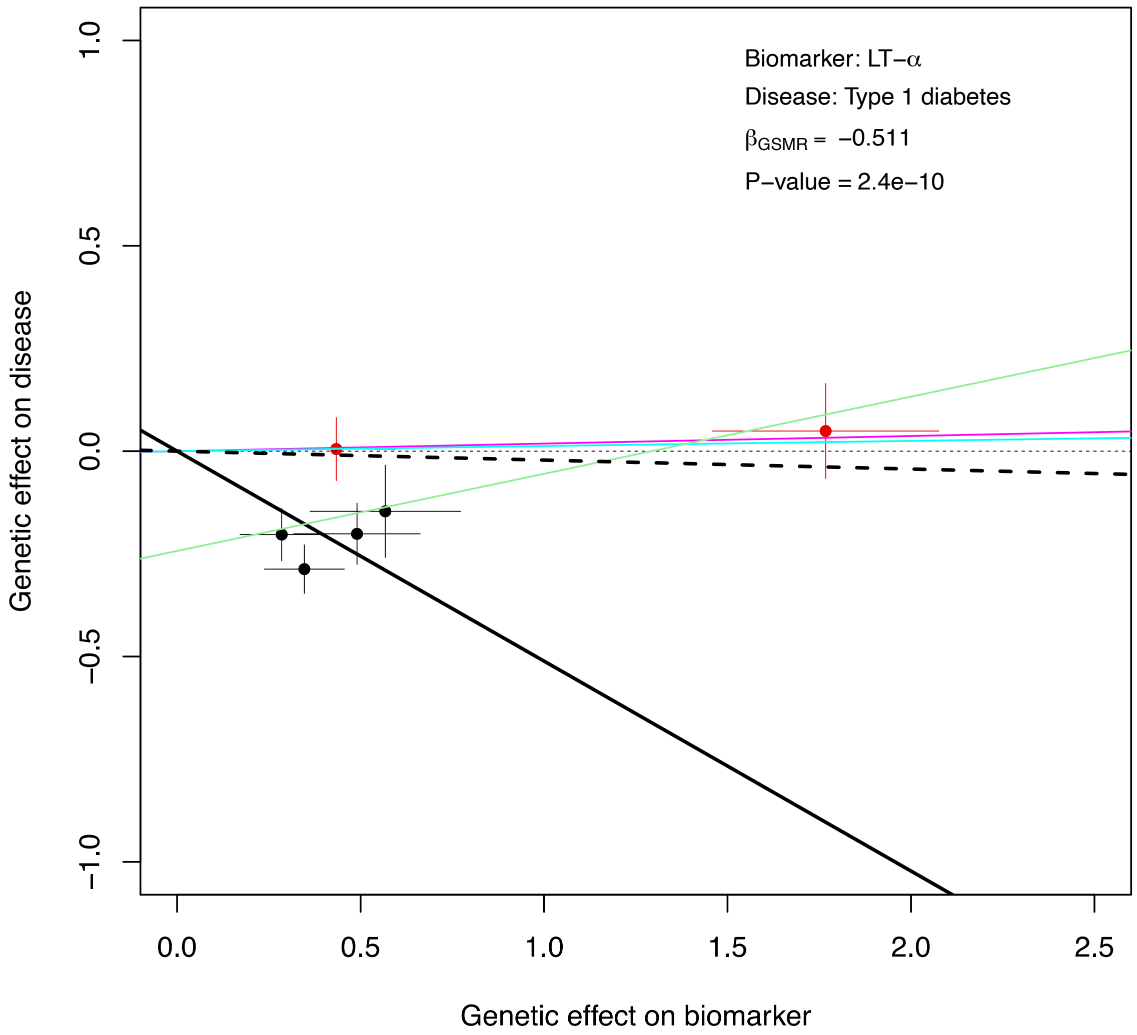

Fig. S3. Causal effect of LT-α on type 1 diabetes. Genetic instruments are shown with their effects on the biomarker plotted against their effects on disease. Black dots (with corresponding 95% CI) denote SNPs that GSMR identifies as being valid, while red dots (with corresponding 95% CI) are identified as statistical outliers by the HEIDI-outlier procedure. Their effect on disease is likely modified by horizontal pleiotropy and they are therefore removed prior to causal estimation. The black solid line denotes the estimate by GSMR which is adopted as the main result (corresponding raw *P* value is shown in figure legend), while the cyan line denotes the estimate by the inverse variance weighted method, the magenta line denotes the estimate by the weighted median method, and the green line denotes the estimate by the MR-Egger method. Note that in the last three, alternative methods, all genetic variants are used in the causal estimation. Finally, the black dashed line denotes the estimate by GSMR with the HEIDI-outlier removal procedure switched off.

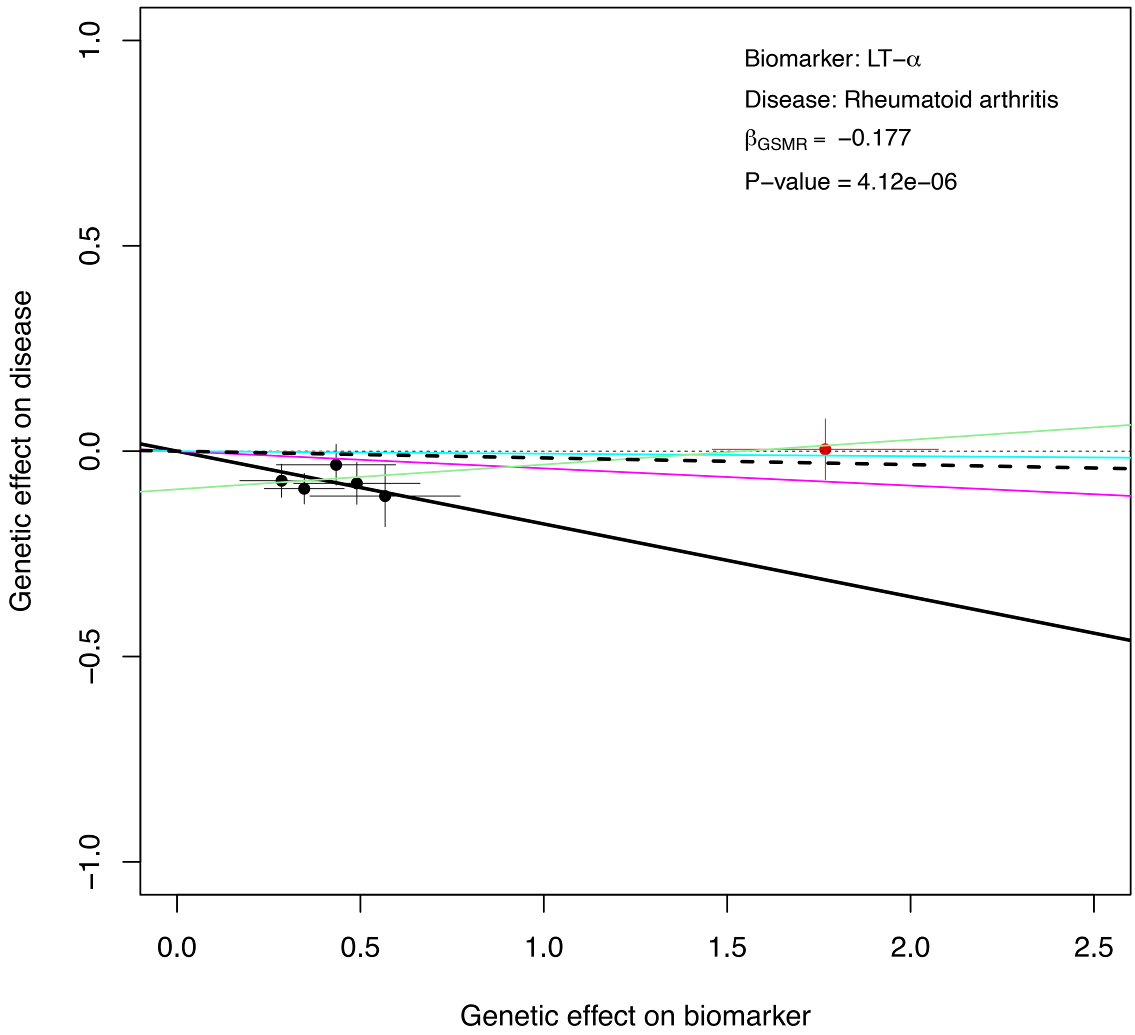

Fig. S4. Causal effect of LT-α on rheumatoid arthritis. Genetic instruments are shown with their effects on the biomarker plotted against their effects on disease. Black dots (with corresponding 95% CI) denote SNPs that GSMR identifies as being valid, while red dots (with corresponding 95% CI) are identified as statistical outliers by the HEIDI-outlier procedure. Their effect on disease is likely modified by horizontal pleiotropy and they are therefore removed prior to causal estimation. The black solid line denotes the estimate by GSMR which is adopted as the main result (corresponding raw *P* value is shown in figure legend), while the cyan line denotes the estimate by the inverse variance weighted method, the magenta line denotes the estimate by the weighted median method, and the green line denotes the estimate by the MR-Egger method. Note that in the last three, alternative methods, all genetic variants are used in the causal estimation. Finally, the black dashed line denotes the estimate by GSMR with the HEIDI-outlier removal procedure switched off.

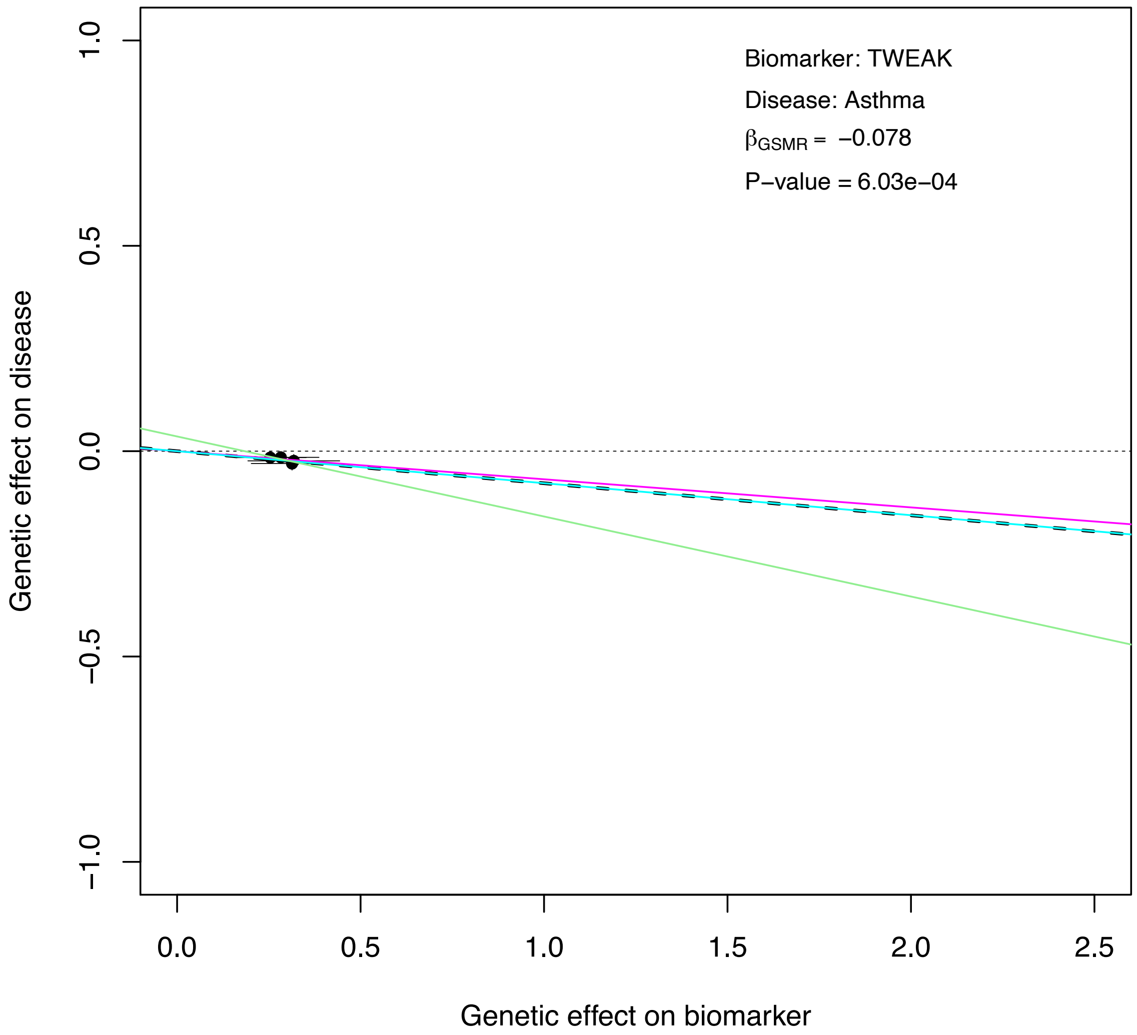

Fig. S5. Causal effect of TWEAK on asthma. Genetic instruments are shown with their effects on the biomarker plotted against their effects on disease. Only four SNPs were available for the MR, wherefore the HEIDI-outlier procedure in GSMR was switched off and all four instruments were assumed to be valid (black dots with corresponding 95% CI). A simple visual inspection of the data also supports this assumption. The black dashed line denotes the estimate by GSMR, with HEIDI switched off, which is adopted as the main result (corresponding raw *P* value is shown in figure legend), while the cyan line denotes the estimate by the inverse variance weighted method, the magenta line denotes the estimate by the weighted median method, and the green line denotes the estimate by the MR-Egger method. Note that in the last three, alternative methods, all genetic variants are used in the causal estimation.

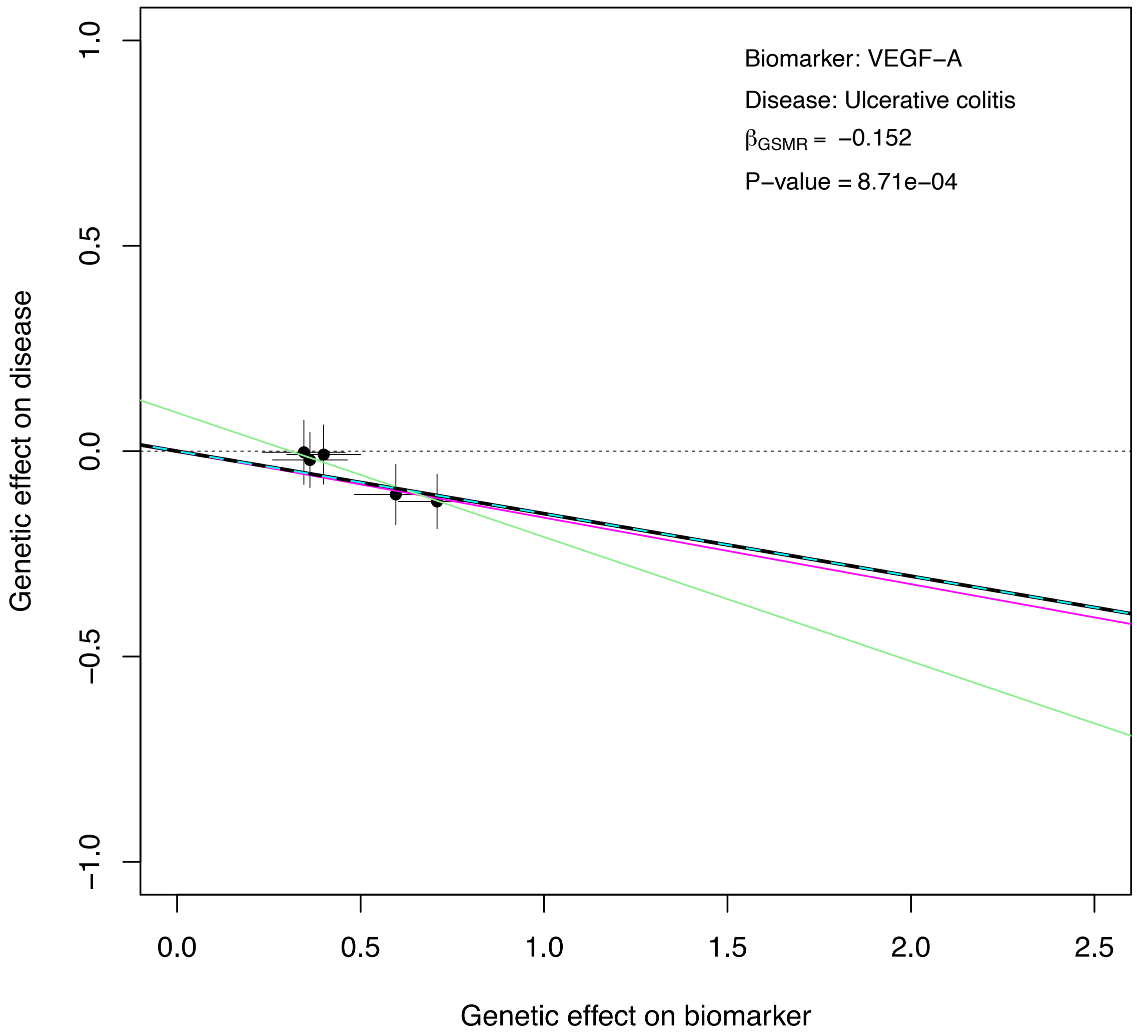

Fig. S6. Causal effect of VEGF-A on ulcerative colitis. Genetic instruments are shown with their effects on the biomarker plotted against their effects on disease. Black dots (with corresponding 95% CI) denote SNPs that GSMR identifies as being valid. No statistical outliers were identified by the HEIDI-outlier procedure. The black solid line denotes the estimate by GSMR which is adopted as the main result (corresponding raw *P* value is shown in figure legend), while the cyan line denotes the estimate by the inverse variance weighted method, the magenta line denotes the estimate by the weighted median method, and the green line denotes the estimate by the MR-Egger method. Note that in the last three, alternative methods, all genetic variants are used in the causal estimation. Finally, the black dashed line denotes the estimate by GSMR with the HEIDI-outlier removal procedure switched off. Note that this line coincides with the black solid line, as no outliers were detected.

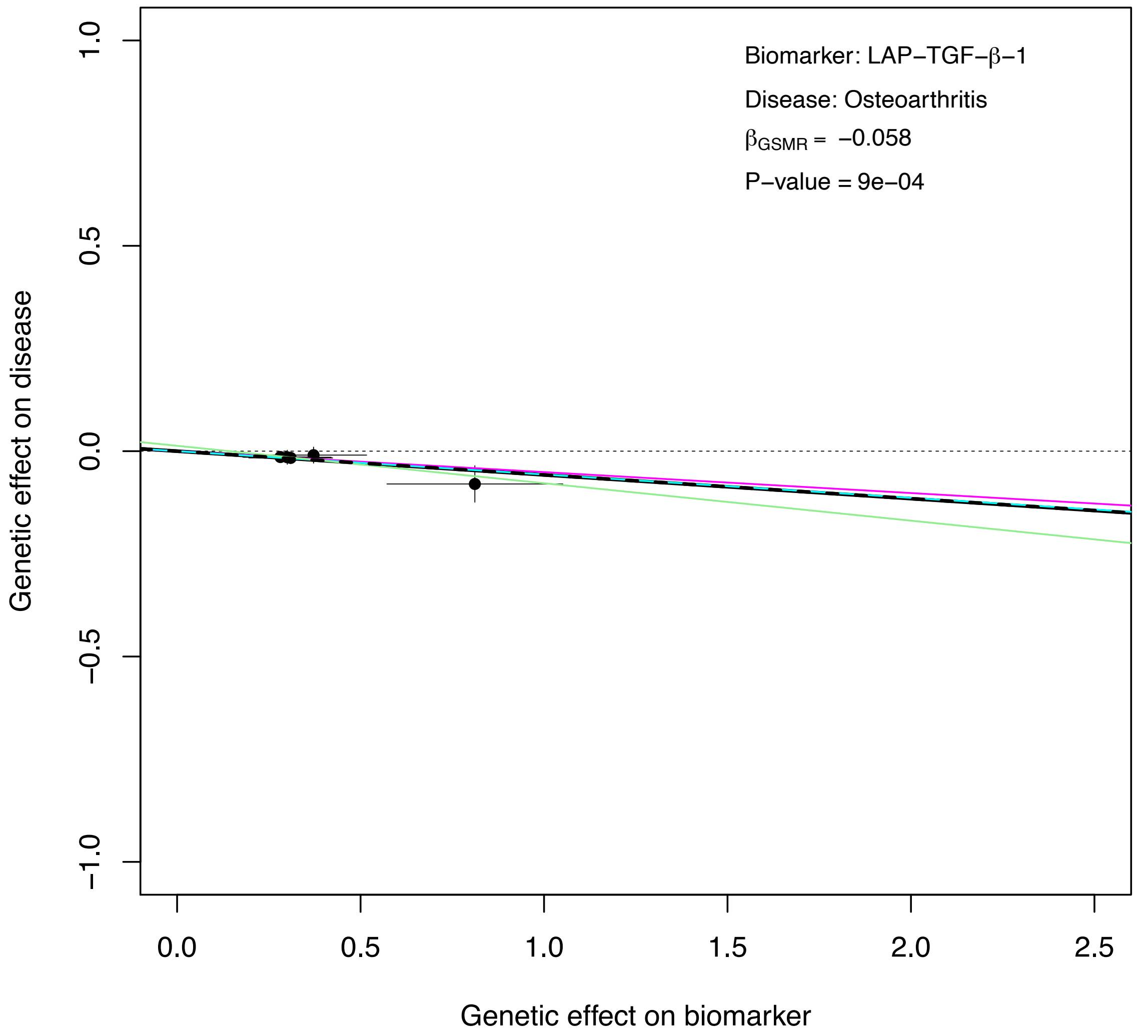

Fig. S7. Causal effect of LAP-TGF-β-1 on osteoarthritis. Genetic instruments are shown with their effects on the biomarker plotted against their effects on disease. Black dots (with corresponding 95% CI) denote SNPs that GSMR identifies as being valid. No statistical outliers were identified by the HEIDI-outlier procedure. The black solid line denotes the estimate by GSMR which is adopted as the main result (corresponding raw *P* value is shown in figure legend), while the cyan line denotes the estimate by the inverse variance weighted method, the magenta line denotes the estimate by the weighted median method, and the green line denotes the estimate by the MR-Egger method. Note that in the last three, alternative methods, all genetic variants are used in the causal estimation. Finally, the black dashed line denotes the estimate by GSMR with the HEIDI-outlier removal procedure switched off. Note that this line coincides with the black solid line, as no outliers were detected.

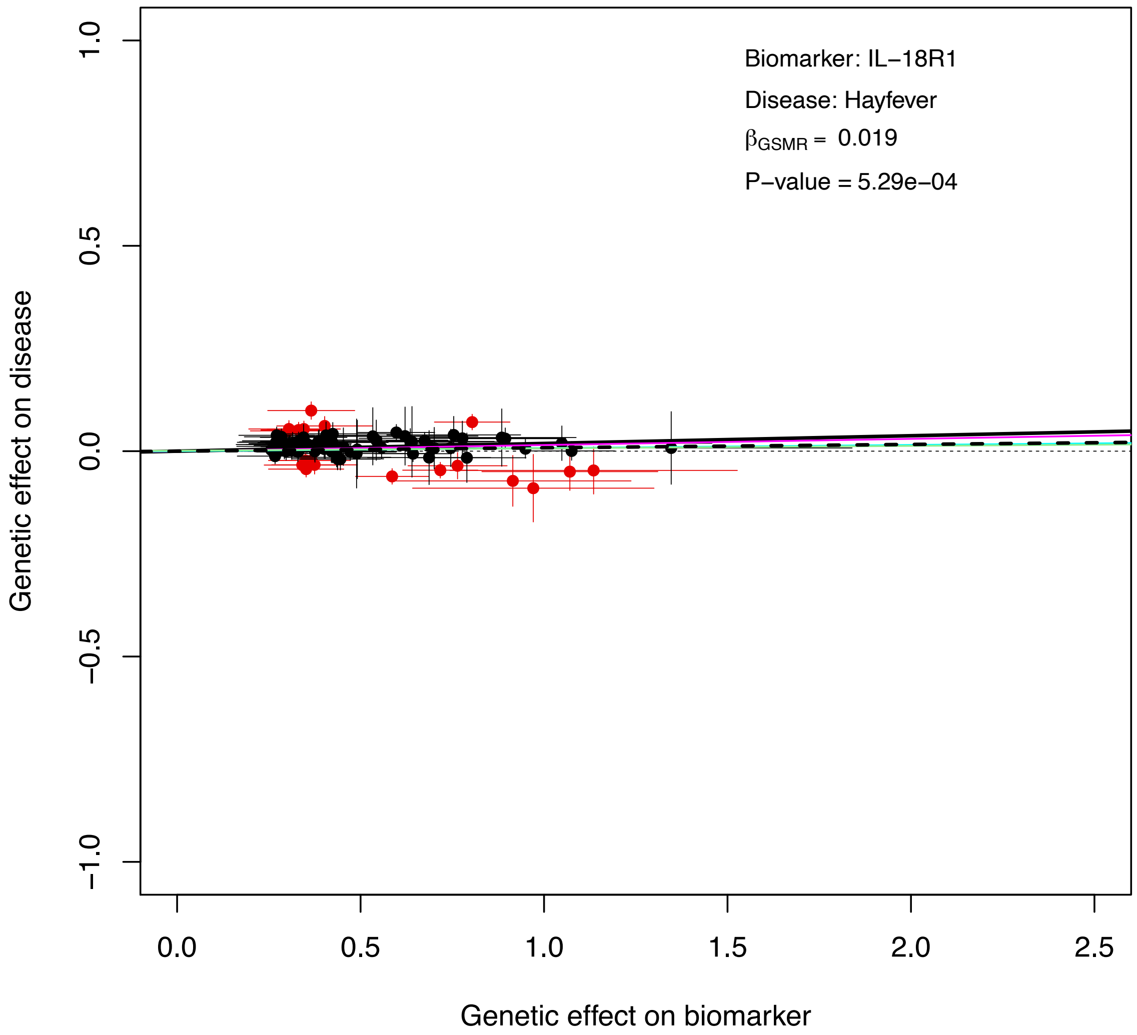

Fig. S8. Causal effect of IL-18R1 on hay fever. Genetic instruments are shown with their effects on the biomarker plotted against their effects on disease. Black dots (with corresponding 95% CI) denote SNPs that GSMR identifies as being valid, while red dots (with corresponding 95% CI) are identified as statistical outliers by the HEIDI-outlier procedure. Their effect on disease is likely modified by horizontal pleiotropy and they are therefore removed prior to causal estimation. The black solid line denotes the estimate by GSMR which is adopted as the main result (corresponding raw *P* value is shown in figure legend), while the cyan line denotes the estimate by the inverse variance weighted method, the magenta line denotes the estimate by the weighted median method, and the green line denotes the estimate by the MR-Egger method. Note that in the last three, alternative methods, all genetic variants are used in the causal estimation. Finally, the black dashed line denotes the estimate by GSMR with the HEIDI-outlier removal procedure switched off.

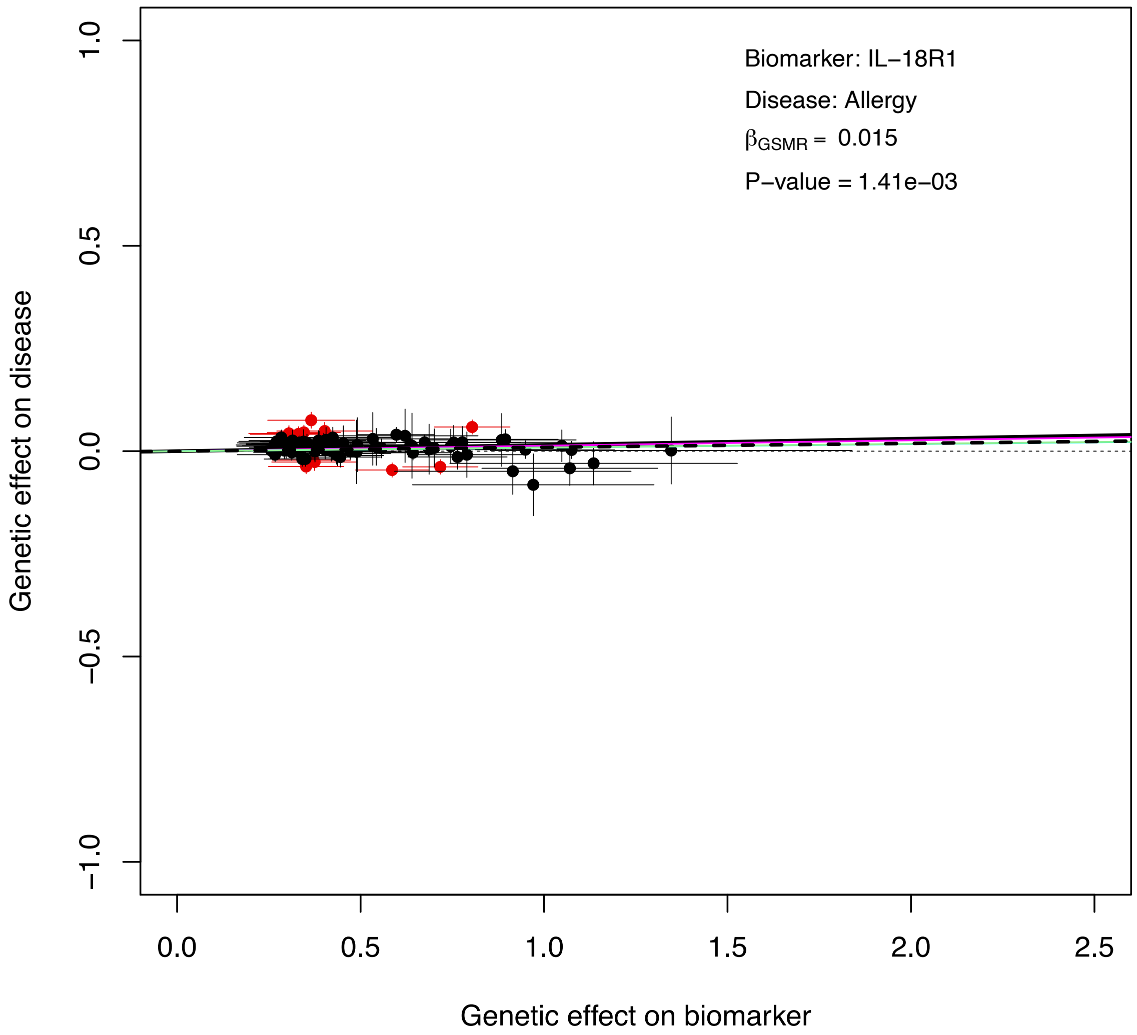

Fig. S9. Causal effect of IL-18R1 on allergy. Genetic instruments are shown with their effects on the biomarker plotted against their effects on disease. Black dots (with corresponding 95% CI) denote SNPs that GSMR identifies as being valid, while red dots (with corresponding 95% CI) are identified as statistical outliers by the HEIDI-outlier procedure. Their effect on disease is likely modified by horizontal pleiotropy and they are therefore removed prior to causal estimation. The black solid line denotes the estimate by GSMR which is adopted as the main result (corresponding raw *P* value is shown in figure legend), while the cyan line denotes the estimate by the inverse variance weighted method, the magenta line denotes the estimate by the weighted median method, and the green line denotes the estimate by the MR-Egger method. Note that in the last three, alternative methods, all genetic variants are used in the causal estimation. Finally, the black dashed line denotes the estimate by GSMR with the HEIDI-outlier removal procedure switched off.

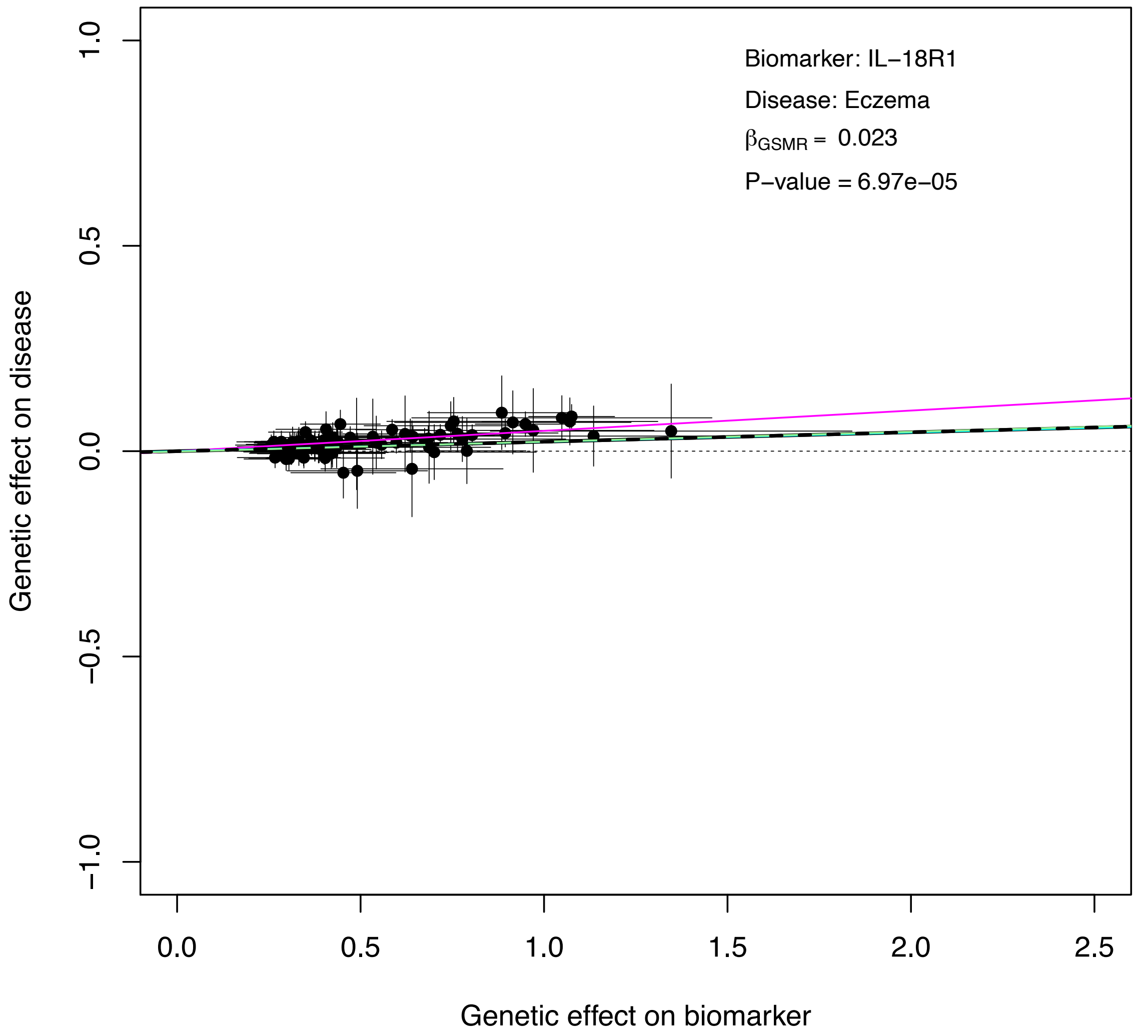

Fig. S10. Causal effect of IL-18R1 on eczema. Genetic instruments are shown with their effects on the biomarker plotted against their effects on disease. Black dots (with corresponding 95% CI) denote SNPs that GSMR identifies as being valid. No statistical outliers were identified by the HEIDI-outlier procedure. The black solid line denotes the estimate by GSMR which is adopted as the main result (corresponding raw *P* value is shown in figure legend), while the cyan line denotes the estimate by the inverse variance weighted method, the magenta line denotes the estimate by the weighted median method, and the green line denotes the estimate by the MR-Egger method. Note that in the last three, alternative methods, all genetic variants are used in the causal estimation. Finally, the black dashed line denotes the estimate by GSMR with the HEIDI-outlier removal procedure switched off. Note that this line coincides with the black solid line, as no outliers were detected.

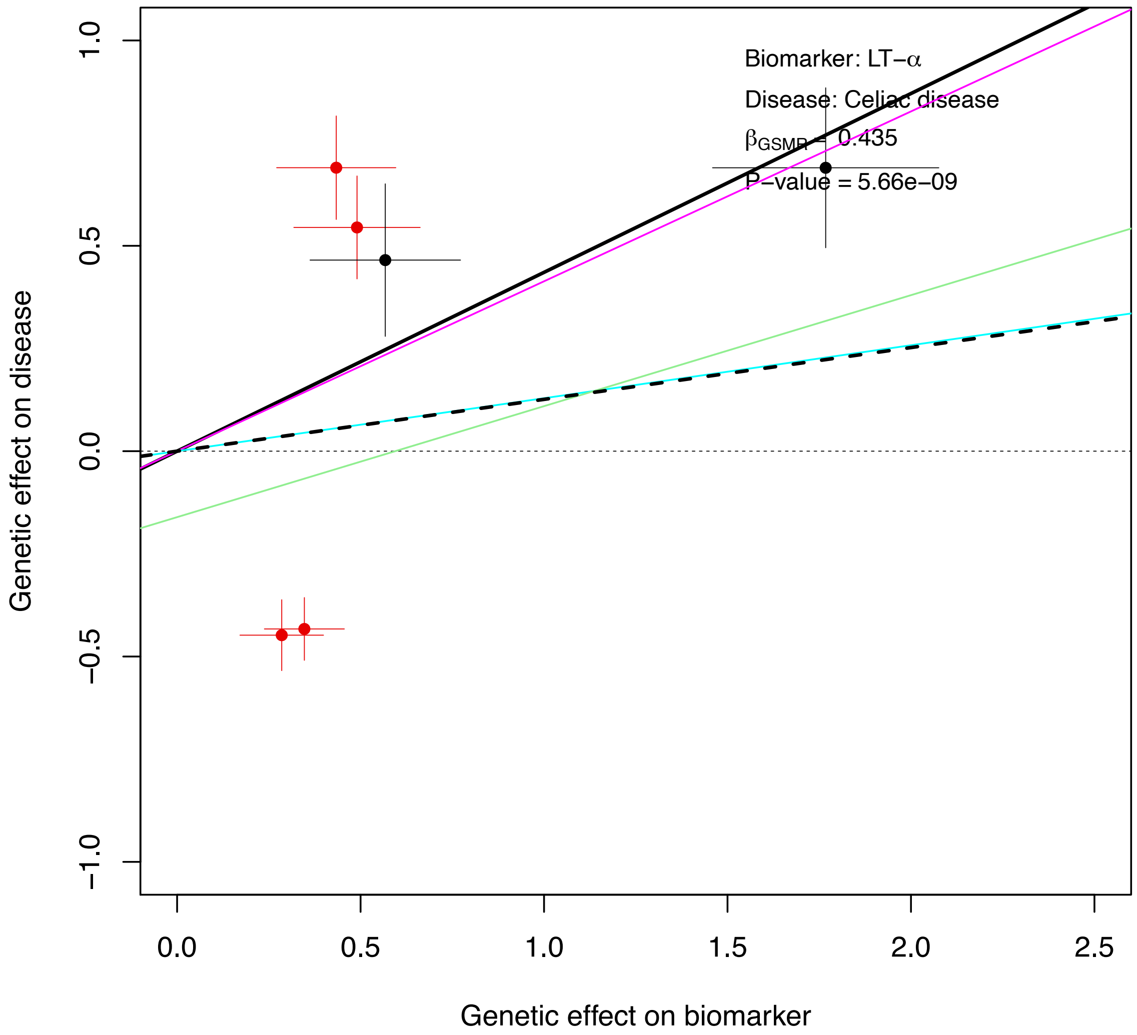

Fig. S11. Causal effect of LT-α on celiac disease. Genetic instruments are shown with their effects on the biomarker plotted against their effects on disease. Black dots (with corresponding 95% CI) denote SNPs that GSMR identifies as being valid, while red dots (with corresponding 95% CI) are identified as statistical outliers by the HEIDI-outlier procedure. Their effect on disease is likely modified by horizontal pleiotropy and they are therefore removed prior to causal estimation. The black solid line denotes the estimate by GSMR which is adopted as the main result (corresponding raw *P* value is shown in figure legend), while the cyan line denotes the estimate by the inverse variance weighted method, the magenta line denotes the estimate by the weighted median method, and the green line denotes the estimate by the MR-Egger method. Note that in the last three, alternative methods, all genetic variants are used in the causal estimation. Finally, the black dashed line denotes the estimate by GSMR with the HEIDI-outlier removal procedure switched off.

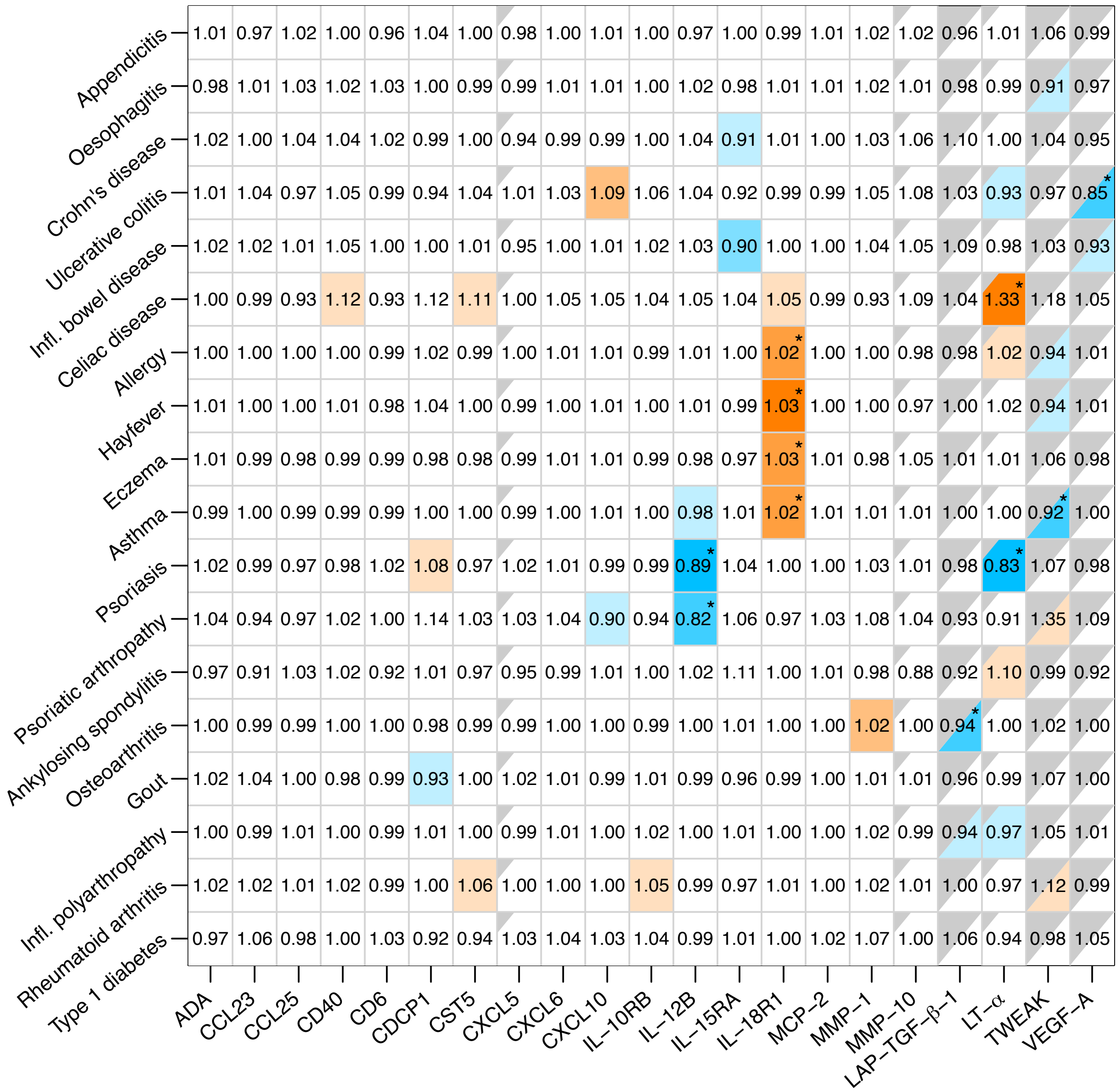

Fig. S12. Causal effect of each biomarker on each inflammatory disease with stricter parameter settings P=10^{-6} and R^2=0.2. Results from the GSMR analysis with stricter parameters settings. The P value threshold is set to P=10^{-6} while the LD threshold is set to R^2=0.2. An asterisk (*) indicates significant effect (FDR<0.05), blue shades denote a protective effect with various significance (raw P value): light blue=[0.01≤P<0.05], medium light blue=[0.001≤P<0.01], medium dark blue=[0.0001≤P<0.001], and dark blue=[P<0.0001], while orange shades denote increased risk with corresponding significance intervals, and white denotes a non-significant result (P≥0.05). Half-colored squares denote estimates with P<0.05, similar to full-colored squares, but, e.g., due to the removal of outlier SNPs, the results are based on less than 4 instruments, which makes the estimates more uncertain. If only four SNPs met the stricter parameter settings, results are indicated by squares with gray upper-left corners. If less than four SNPs met the stricter thresholds, results are indicated by half-grayed squares. In both these situations (i.e.,≤ 4 SNPs), the HEIDI-outlier removal procedure was switched off.

	ADA	CCL23	CCL25	CD40	CD6	CDCP1	CST5	CXCL5	CXCL6	CXCL10	IL−10RB	IL−12B	IL−15RA	IL−18R1	MCP−2	MMP−1	MMP−10	LAP−TGF−β−1	LT−α	TWEAK	VEGF−A
Appendicitis	1.01	0.98	1.00	0.98	0.95	0.96	1.00	0.98	0.96	1.01	1.01	0.98	1.00	1.00	1.00	0.99	NA	NA	1.00	NA	0.98
Oesophagitis	0.99	1.00	1.02	1.00	1.02	0.95	1.02	1.01	1.00	1.02	1.01	1.02	0.99	1.00	1.01	1.03	NA	NA	0.99	NA	0.97
Crohn's disease	1.05	0.99	1.02	0.97	1.02	1.12	1.03	0.94	0.99	1.01	0.98	1.05	0.95	1.02	1.00	1.03	NA	NA	0.97	NA	0.96
Ulcerative colitis	0.98	1.01	0.95	1.04	0.95	0.94	1.07	1.02	1.03	1.09	1.07	1.04	0.92	1.02	0.99	1.04	NA	NA	0.96	NA	0.86*
Infl. bowel disease	1.03	1.00	0.99	0.99	0.99	1.09	1.04	0.96	0.99	1.02	1.02	1.05	0.94	1.01	0.99	1.03	NA	NA	0.98	NA	0.93
Celiac disease	0.97	1.02	0.95	1.05	0.94	1.00	1.09	1.00	1.00	1.05	1.04	1.00	0.98	1.06*	1.00	0.95	NA	NA	0.98	NA	1.05
Allergy	1.01	0.99	1.00	1.02	0.98	1.01	0.99	1.00	1.01	1.01	1.00	1.01	0.99	1.02*	1.00	1.00	NA	NA	1.03	NA	1.01
Hayfever	1.01	0.99	1.00	1.02	0.98	1.00	1.00	0.99	1.00	1.00	1.01	1.00	0.99	1.01	1.00	1.00	NA	NA	1.02	NA	1.01
Eczema	1.01	0.99	0.98	0.97	0.98	0.99	1.00	0.99	1.02	1.00	1.00	0.98	0.96	1.03*	1.01	0.97	NA	NA	0.99	NA	0.98
Asthma	0.99	1.02	0.99	1.01	0.99	0.99	1.01	0.99	1.00	1.00	0.99	0.99	1.03	1.01	1.01	1.01	NA	NA	1.00	NA	1.00
Psoriasis	1.03	1.00	0.98	0.92*	1.02	1.03	0.97	1.03	0.99	0.98	0.98	0.82*	1.01	1.00	1.00	1.02	NA	NA	1.00	NA	0.98
Psoriatic arthropathy	1.05	0.95	0.95	0.96	0.96	0.95	1.00	1.04	1.00	0.91	0.90	0.73*	1.04	1.01	1.02	1.06	NA	NA	1.01	NA	1.09
Ankylosing spondylitis	0.97	1.03	1.06	1.01	0.91	1.02	0.97	0.94	1.00	1.02	0.95	1.02	1.11	1.01	1.00	0.96	NA	NA	0.98	NA	0.93
Osteoarthritis	0.99	0.97	0.99	1.01	0.99	0.99	0.99	0.99	1.00	1.00	1.00	0.99	1.01	1.00	1.00	1.01	NA	NA	1.00	NA	1.00
Gout	1.04	1.04	1.01	0.99	0.99	0.95	0.99	1.02	1.00	0.98	1.02	1.00	0.95	1.00	1.00	1.01	NA	NA	1.00	NA	0.99
Infl. polyarthropathy	0.99	0.99	1.01	1.00	1.00	1.03	1.00	1.00	1.00	0.99	1.02	1.00	0.97	1.01	1.00	1.02	NA	NA	0.99	NA	1.01
Rheumatoid arthritis	1.00	0.99	1.00	1.02	0.97	1.02	1.03	1.00	1.02	0.99	1.04	0.98	0.98	1.00	1.01	1.01	NA	NA	1.00	NA	1.00
Type 1 diabetes	0.96	1.02	0.99	1.00	1.03	0.96	0.98	1.03	1.06	1.05	1.04	1.00	1.02	1.00	1.03	1.07	NA	NA	0.99	NA	1.05

Fig. S13. Causal effect of each biomarker on each inflammatory disease with stricter parameter settings P=5×10^{-8} and R^2=0.6. Results from the GSMR analysis with stricter parameters settings. The P value threshold is set to P=5×10^{-8} while the LD threshold is set to R^2=0.6. If only one SNP was left after filtering, GSMR was unable to perform a causal estimation, which is indicated by "NA". An asterisk (*) indicates significant effect (FDR<0.05), blue shades denote a protective effect with various significance (raw P value): light blue=[0.01≤P<0.05], medium light blue=[0.001≤P<0.01], medium dark blue=[0.0001≤P<0.001], and dark blue=[P<0.0001], while orange shades denote increased risk with corresponding significance intervals, and white denotes a non-significant result (P≥0.05). Half-colored squares denote estimates with P<0.05, similar to full-colored squares, but, e.g., due to the removal of outlier SNPs, the results are based on less than 4 instruments, which makes the estimates more uncertain. If only four SNPs met the stricter parameter settings, results are indicated by squares with gray upper-left corners. If less than four SNPs met the stricter thresholds, results are indicated by half-grayed squares. In both these situations (i.e., ≤4 SNPs), the HEIDI-outlier removal procedure was switched off.

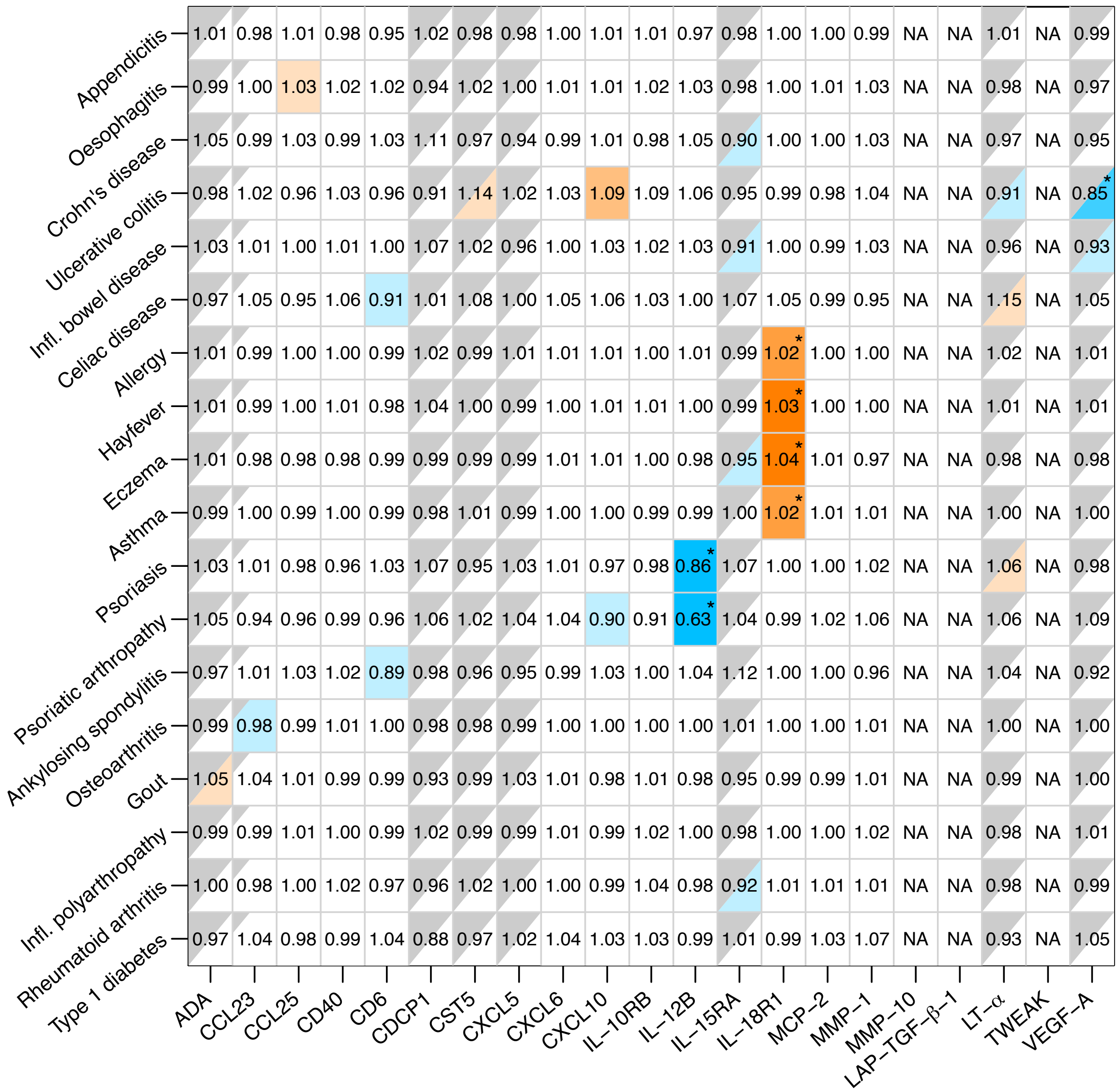

Fig. S14. Causal effect of each biomarker on each inflammatory disease with stricter parameter settings P=5×10^{-8} and R^2=0.2. Results from the GSMR analysis with stricter parameters settings. The P value threshold is set to P=5×10^{-8} while the LD threshold is set to R^2=0.2. If only one SNP was left after filtering, GSMR was unable to perform a causal estimation, which is indicated by "NA". An asterisk (*) indicates significant effect (FDR<0.05), blue shades denote a protective effect with various significance (raw P value): light blue=[0.01≤P<0.05], medium light blue=[0.001≤P<0.01], medium dark blue=[0.0001≤P<0.001], and dark blue=[P<0.0001], while orange shades denote increased risk with corresponding significance intervals, and white denotes a non-significant result (P≥0.05). Half-colored squares denote estimates with P<0.05, similar to full-colored squares, but, e.g., due to the removal of outlier SNPs, the results are based on less than 4 instruments, which makes the estimates more uncertain. If only four SNPs met the stricter parameter settings, results are indicated by squares with gray upper-left corners. If less than four SNPs met the stricter thresholds, results are indicated by half-grayed squares. In both these situations (i.e., ≤4 SNPs), the HEIDI-outlier removal procedure was switched off.

Diseases	UKB data-fields[1] (UKB coding)	Type of data (disease classification)	Cases (total UKB)	Cases (after filtering)
Oesophagitis	41202/41204/40001/40002 (K20)	Register data[2] (ICD-10)	12295	8984
	41203/41205 (5301)	Register data[2] (ICD-9)		
	20002 (1139)	Self-reported[3] (UKB coding)		
Ankylosing spondylitis	41202/41204/40001/40002 (M08.1, M45)	Register data[2] (ICD-10)	1731	1265
	41203/41205 (7200)	Register data[2] (ICD-9)		
	20002 (1313)	Self-reported[3] (UKB coding)		
Psoriatic arthropathy	41202/41204/40001/40002 (M07.1, M07.2, M07.3)	Register data[2] (ICD-10)	1380	1025
	41203/41205 (6960)	Register data[2] (ICD-9)		
	20002 (1477)	Self-reported[3] (UKB coding)		
Psoriasis	41202/41204/40001/40002 (L40)	Register data[2] (ICD-10)	7484	5542
	41203/41205 (6961)	Register data[2] (ICD-9)		
	20002 (1453)	Self-reported[3] (UKB coding)		
Gout	41202/41204/40001/40002 (M10)	Register data[2] (ICD-10)	9348	6830
	41203/41205 (274)	Register data[2] (ICD-9)		
	20002 (1466)	Self-reported[3] (UKB coding)		
Inflammatory polyarthropathy	41202/41204/40001/40002 (M05-M14)	Register data[2] (ICD-10)	22868	16302
	41203/41205 (714)	Register data[2] (ICD-9)		
Appendicitis	41202/41204/40001/40002 (K35-K37)	Register data[2] (ICD-10)	5401	3798
	41205/41203 (540-542)	Register data[2] (ICD-9)		
	20002 (1502)	Self-reported[3] (UKB coding)		
Celiac disease	41202/41204/40001/40002 (K90.0)	Register data[2] (ICD-10)	2148	1616
	41205/41203 (5790)	Register data[2] (ICD-9)		
	21068 (3262),	Self-reported[3] (UKB coding)		
Allergy	20002 (1387,1385,1386,1668,1562,1563)	Self-reported[3] (UKB coding)	37891	27826
Eczema	41202/41204/40001/40002 (L2, L3)	Register data[2] (ICD-10)	17620	12942
	41203/41205 (691, 692)	Register data[2] (ICD-9)		
Hay fever	41202/41204/40001/40002 (J301-J304)	Register data[2] (ICD-10)	31790	23229
	41203/41205 (477)	Register data[2] (ICD-9)		
	20002 (1387), 22126 (1)	Self-reported[3] (UKB coding)		
Asthma	41202/41204/40001/40002 (J45)	Register data[2] (ICD-10)	66492	47628
	41203/41205 (493),	Register data[2] (ICD-9)		
	20002 (1111), 6152(8)	Self-reported[3,4] (UKB coding)		
Crohn's Disease	41202/41204/40001/40002 (K51)	Register data[2] (ICD-10)	4725	3424
	20002 (1463)	Self-reported[3] (UKB coding)		
Ulcerative Colitis	41202/41204/40001/40002 (K50)	Register data[2] (ICD-10)	2459	1771

	20002 (1462)	Self-reported[3] (UKB coding)		
Inflammatory Bowel Disease	41202/41204/40001/40002 (K50-K51)	Register data[2] (ICD-10)	6758	4898
	20002 (1461-1463)	Self-reported[3] (UKB coding)		
Osteoarthritis	41202/41204/40001/40002 (M15-M18, M19.0, M19.8, M19.9)	Register data[2] (ICD-10)	79196	58587
	41203/41205 (715.0, 715.1, 715.3,715.8, 715.9)	Register data[2] (ICD-9)		
	20002 (1465)	Self-reported[3] (UKB coding)		
Rheumatoid arthritis	41202/41204/40001/40002 (M05, M06)	Register data[2] (ICD-10)	8592	6097
	41203/41205 (7140)	Register data[2] (ICD-9)		
	20002 (1464)	Self-reported[3] (UKB coding)		
Type 1 diabetes	41202/41204/40001/40002 (E10)	Register data[2] (ICD-10)	3807	2577
	20002 (1222)	Self-reported[3] (UKB coding)		

Table S1. Identification of inflammatory diseases in UK Biobank (UKB)

1) UKB data-field is the name used in the UKB database and UKB coding is how the diagnoses are coded for respective data-fields. This coding is represented either by an ICD-9 or ICD-10 code (for register data), or an internal UKB code (for self-reported data).
2) Data from the registers represent in-patient hospital and cause-of-death data. Most diseases have diagnoses that are coded according to ICD-10 and some also according to ICD-9.
3) Self-reported data are either from verbal interview (data-field 20002), or touch-screen questionnaire (data-fields: 6152, 22126). The coding for theses variables represents how they are coded in the UKB database.
